# Structural characterization of quaternary selenites of tungsten(VI), *A*_2_W_3_SeO_12_ (*A* = NH_4_, Cs, Rb, K or Tl)

**DOI:** 10.1107/S2056989021002735

**Published:** 2021-03-19

**Authors:** Vineela Balisetty, Kanamaluru Vidyasagar

**Affiliations:** a Department of Chemistry, Indian Institute of Technology Madras, Chennai 600 036, India

**Keywords:** single-crystal X-ray structure, quaternary selenite, tungsten

## Abstract

The quaternary *A*_2_W_3_SeO_12_ (*A* = NH_4_, Cs, Rb, K or Tl) selenites have been prepared in the form of single crystals by hydro­thermal and novel solid-state reactions. They were characterized by X-ray diffraction, thermal and spectroscopic studies. All of them have a hexa­gonal tungsten oxide (HTO) related [W_3_SeO_12_]^2−^ anionic framework with pyramidally coordinated Se^4+^ ions. The known *A*_2_W_3_SeO_12_ (*A* = NH_4_, Cs or Rb) compounds are isostructural with the Cs_2_W_3_TeO_12_ compound and have a non-centrosymmetric layered structure containing intra-layer Se—O bonds. The new compound K_2_W_3_SeO_12_(α) is isostructural with the K_2_W_3_TeO_12_ compound and has a centrosymmetric three-dimensional structure containing inter­layer Se—O bonds. It is inferred that the new Tl_2_W_3_SeO_12_ compound has the same three-dimensional structure as K_2_W_3_SeO_12_(α).

## Abstract 2

The quaternary *A*_2_W_3_SeO_12_ (*A* = NH_4_, Cs, Rb, K or Tl) selenites have been prepared in the form of single crystals by hydro­thermal and novel solid-state reactions. They were characterized by X-ray diffraction, thermal and spectroscopic studies. All of them have a hexa­gonal tungsten oxide (HTO) related [W_3_SeO_12_]^2−^ anionic framework with pyramidally coordinated Se^4+^ ions.

## Chemical context   

Non-centrosymmetric (NCS) compounds are widely studied as they have potentially useful symmetry-dependent properties such as piezoelectricity, ferroelectricity and second-order non-linear optical (NLO) behaviour (Halasyamani & Poeppelmeier 1998 ▸). Many crystalline selenites and tellurites containing *d* ^0^ transition-metal ions such as V^5+^, Mo^6+^, W^6+^ are non-centrosymmetric compounds. The solid-state chemistry of these oxides is inter­esting from the point of view of both structural diversity and second harmonic generation (SHG) activity. They have two types of second-order Jahn–Teller (SOJT) distortion. One is the distorted octa­hedral coordination of the *d* ^0^ transition-metal ion and the other is pyramidal, disphenoidal and square-pyramidal coordinations of Se^4+^ and Te^4+^, which have stereoactive lone pairs. Both SOJT distortions lead to acentric coordination environments that are conducive for NCS structures (Halasyamani 2004 ▸). For example, Cs_2_Mo_3_TeO_12_ (Vidyavathy Balraj & Vidyasagar, 1998 ▸) and YVSe_2_O_8_ (Kim *et al.*, 2014 ▸) have non-centrosymmetric layered structures with these SOJT distortions and exhibit SHG activity. It needs to be mentioned that quaternary selenites and tellurites containing *d* ^0^ transition-metal ions, such as YVTe_2_O_8_ (Kim *et al.*, 2014 ▸), are also known to have centrosymmetric structures and exhibit no SHG activity.

*A*_2_Mo_3_SeO_12_ (*A* = NH_4_, Cs, Rb, Tl) (Harrison *et al.*, 1994 ▸; Dussack *et al.*, 1996 ▸; Chang *et al.*, 2010 ▸), *A*_2_W_3_SeO_12_ (*A* = NH_4_, Cs, Rb, K) (Harrison *et al.*, 1995 ▸; Huang *et al.*, 2014*a* ▸,*b* ▸) and Na_2_W_3_SeO_12_·2H_2_O (Nguyen & Halasyamani 2013 ▸), *A*_2_Mo_3_TeO_12_ (*A* = Cs, NH_4_) (Vidyavathy Balraj & Vidyasagar 1998 ▸), *A*_2_W_3_TeO_12_ (*A* = K, Rb and Cs) (Goodey *et al.*, 2003 ▸; Zhao *et al.*, 2015 ▸) are the 14 quaternary selenites and tellurites of hexa­valent molybdenum and tungsten that have hexa­gonal tungsten oxide (HTO) related [*M*_3_ *X*O_12_]^2−^ (*M* = Mo, W; *X* = Se, Te) anionic frameworks with pyramidally coordinated Se^4+^ and Te^4+^ ions. The single-crystal X-ray structures were determined for all except for the *A*_2_W_3_SeO_12_ (*A* = NH_4_, Cs, Rb) compounds, which were synthesized in polycrystalline form by the hydro­thermal method; the structures of the (NH_4_)_2_W_3_SeO_12_ and Cs_2_W_3_SeO_12_ compounds were determined by powder neutron diffraction (Harrison *et al.*, 1995 ▸). K_2_W_3_TeO_12_ has a centrosymmetric three-dimensional structure (Goodey *et al.*, 2003 ▸), whereas all of the others exhibit a non-centrosymmetric two-dimensional structure and show SHG response. It is noteworthy that the tellurites were synthesized by both hydro­thermal and solid-state reactions, whereas the selenites were synthesized only by the hydro­thermal method.

Ag_2_Mo_3_SeO_12_ (Ling & Albrecht-Schmitt 2007 ▸), Li_2_Mo_3_TeO_12_ (Oh *et al.*, 2018 ▸) and *A*_4_Mo_6_Te_2_O_24_·6H_2_O (*A* = Rb, K) (Vidyavathy Balraj & Vidyasagar 1998 ▸) compounds are quaternary selenite and tellurites of molybdenum, whose [Mo_3_ *X*O_12_]^2−^ (*X* = Se, Te) anionic framework structures are not related to HTO. They have centrosymmetric layered and zero-dimensional structures and contain pyramidally coordinated Se^4+^ and pyramidally and disphenoidally coordinated Te^4+^ ions.

In this context, the structural characterization of new and known quaternary *A*_2_W_3_SeO_12_ (*A* = NH_4_, Cs, Rb, K, Tl) selenites of tungsten(VI) by single-crystal X-ray diffraction was considered necessary for their complete structural study and, therefore, was undertaken. This report is concerned with crystal growth by solid-state reactions and structural characterization of the known compounds *A*_2_W_3_SeO_12_ [*A* = NH_4_ (**1**), Cs (**2**) and Rb (**3**)] and new compounds K_2_W_3_SeO_12_ (**4α**) and Tl_2_W_3_SeO_12_ (**5**).

## Structural commentary   

The structures of compounds **1**–**5** are of two types, which contain a hexa­gonal tungsten oxide (HTO) related [W_3_SeO_12_]^2−^ anionic framework. (NH_4_)_2_W_3_SeO_12_ (**1**), Cs_2_W_3_SeO_12_ (**2**) and Rb_2_W_3_SeO_12_ (**3**) crystallize in the *P*6_3_ space group and have the structure of Cs_2_W_3_TeO_12_ (Zhao *et al.*, 2015 ▸). They contain ammonium/caesium/rubidium ions between non-centrosymmetric HTO-related [W_3_SeO_12_]^2−^ layers, which have only intra-layer type Se—O bonds. The absolute structure configuration of the rubidium compound (**3**) is the inverse of that of the ammonium (**1**) and caesium (**2**) compounds.

**Figure 1:**
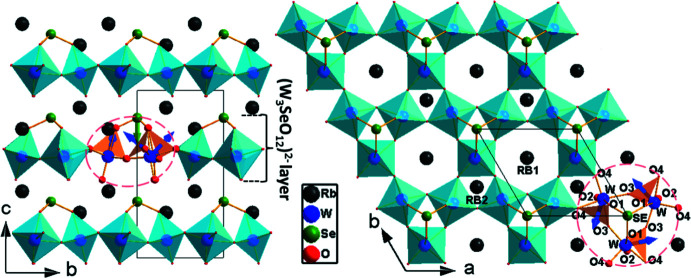
Polyhedral representation of (left) the unit-cell structure viewed along the *a* axis and (right) a (W_3_SeO_12_)^2−^ layer along with the Rb^+^ counter-cations, viewed along the *c* axis, of Rb_2_W_3_SeO_12_ (**3**). A W_3_O_15_ moiety with a pyramidal selenium atom is indicated by a dashed red line and the net dipole directions of the WO_6_ octa­hedra and pyramidal SeO_3_ are shown.

As an illustrative example, the structure of Rb_2_W_3_SeO_12_ (**3**) is discussed. Its asymmetric unit content of Rb_2/3_WTe_1/3_O_4_ has two, one, one and four crystallographically distinct rubidium, tungsten, selenium and oxygen atoms, respectively. The tungsten atom is octa­hedrally coordinated to the apical O1 and O2 atoms and two each of equatorial O3 and O4 atoms (Fig. 1 ▸). The WO_6_ octa­hedron resides near the threefold rotation axis located at the Wyckoff site 2*a* and shares its two *cis* O3 equatorial oxygen atoms with two such octa­hedra to form a W_3_O_15_ moiety. Such trinuclear moieties are connected to one another through sharing of equatorial O4 atoms, forming a hexa­gonal–tungsten–oxide (HTO) layer of composition WO_4_ or W_3_O_12_. In other words, the HTO layer of WO_4_ is formed from the sharing of four equatorial O3 and O4 atoms of every WO_6_ octa­hedron with four such octa­hedra. The HTO layer of WO_4_ has three-ring holes made of either O3 or O4 atoms and six-ring holes made of alternating O3 and O4 atoms. The selenium atom resides on a threefold rotation axis located at the 2*a* site and has a pyramidal coordination of *C*_3*V*_ symmetry, with three equivalent Se—O1 bonds. Thus, only three-ring holes of O3 are capped on one side of the layer, by bonding of the selenium atom to apical O1 oxygen atoms, to give rise to an asymmetric (W_3_SeO_12_)^2−^ layer. These layers are stacked, as shown in Fig. 1 ▸, along the crystallographic *c-*axis direction in the *ABAB*… fashion because adjacent layers are rotated with respect to each other such that the six-ring hole of one layer is above the uncapped three-ring hole of the next layer. As the other apical oxygen O2 atoms are not bonded to selenium, the Se—O bonding is described as intra-layer bonding and, therefore, the structure is two-dimensional. The pyramidal SeO_3_ moieties and the lone-pair of electrons of Se^4+^ are respectively parallel and perpendicular to the HTO layers of WO_4_. The selenites **1**–**5** of the present study are found to contain the same staggered stacking of the HTO-related WO_4_ layers.

**Figure 2:**
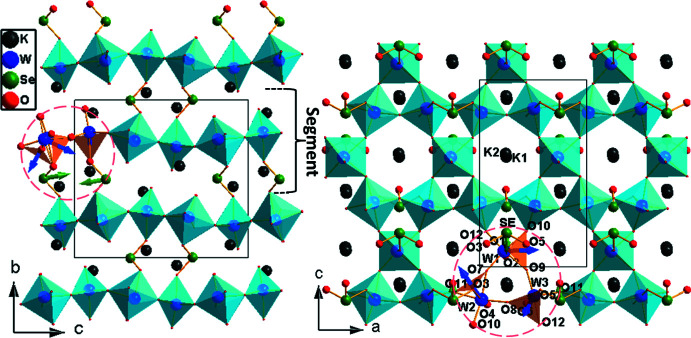
Polyhedral representation of (left) the unit-cell structure viewed along the −*a* axis and (right) a segment of the (W_3_SeO_12_)^2−^ structure along with the K^+^ counter-cations, viewed along the −*b* axis, of K_2_W_3_SeO_12_ (**4α**). A W1W2W3O_15_ moiety with pyramidal selenium is indicated by a dashed red line and the net dipole directions of octa­hedral WO_6_ and pyramidal SeO_3_ are shown.

K_2_W_3_SeO_12_ (**β**) was reported (Huang *et al.*, 2014*a* ▸,*b* ▸) to be obtained under hydro­thermal conditions and found to contain similar non-centrosymmetric HTO-related [W_3_SeO_12_]^2−^ layers with intra-layer Se—O bonds. On the other hand, K_2_W_3_SeO_12_ (**4α**) of the present study was prepared by solid-state reaction and is isostructural with the reported K_2_W_3_TeO_12_ (Goodey *et al.*, 2003 ▸). Its centrosymmetric, three-dimensional HTO-related [W_3_SeO_12_]^2−^ framework contains inter-layer Se—O bonds (Fig. 2 ▸) and its asymmetric unit has one formula unit. The three W1—W3 atoms are octa­hedrally coordinated to six apical O1–O6 and six equatorial O7–O12 oxygen atoms. The three WO_6_ octa­hedra in the trinuclear W1W2W3O_15_ moieties share equatorial O7–O9 oxygen atoms and these moieties are connected to one another through the other equatorial O10–O12 oxygen atoms to form the WO_4_ layer. The Se atom forms inter­layer Se—O bonds, by bonding to the apical O7, O10 and O12 oxygen atoms of W1W2W3O_15_ moieties of adjacent HTO layers (Fig. 2 ▸) and thus the [W_3_SeO_12_]^2−^ framework is three-dimensional in nature.

Tl_2_W_3_SeO_12_ (**5**) has an ortho­rhom­bic unit cell with *a*_o_ = 11.5962 (10) Å, *b*_o_ = 12.7206 (5) Å and *c*_o_ = 7.2362 (9) Å. The structure refinements in the non-centrosymmetric *Pna*2_1_ and centrosymmetric *Pnam* space groups led to the respective structure agreement factor values of 6.37% and 15.98%; the structure refinements were unsatisfactory, mostly due to X-ray absorption. Its single crystal X-ray structure solution model is found to be same as the three-dimensional structure of K_2_W_3_SeO_12_ (**4α**) and its observed powder XRD pattern (Figure S1b in the supporting information) agrees reasonably with the one simulated on the basis of this model structure. Moreover, the powder XRD patterns and unit-cell parameters of these two compounds are similar. The ortho­rhom­bic unit-cell parameters of the thallium (**5**) compound are related to the monoclinic unit-cell parameters of the potassium (**4** ***α***) compound as follows: *a*_o_ ≃ *b*_m_, *b*_o_ ≃ *c*_m_, *c*_o_ ≃ *a*_m_ and *α*_o_ = 90° ≃ *β*_m_. The single-crystal X-ray data for the thallium compound (**5**) in the centrosymmetric *P*2_1_/*n* space group, corresponding to the potassium compound (**4** ***α***), led to the same structure model and a high value of 19.18% for the structure-agreement factor. It is inferred from these observations that the Tl_2_W_3_SeO_12_ compound (**5**) has the same three-dimensional structure as K_2_W_3_SeO_12_ (**4α**).

In the structurally characterized compounds **1**–**4α** of the present study, the WO_6_ octa­hedra have *C*_3_ distortion as three W—O bonds are <1.9 Å long and their three *trans* W—O bonds are >1.9 Å long; the values of WO_6_ intra­octa­hedral distortions (Halasyamani 2004 ▸), Δ_*d*_, are calculated to be in the 0.73–0.86 range (Table S1). The Se^4+^ ions have pyramidal coordination. The W—O and Se—O bond-length values are in the 1.703 (17)–2.184 (9) Å and 1.695 (10)–1.739 (10) Å ranges, respectively. The ammonium and alkali metal ions are found to be six- to nine-coordinated (Figure S2), when the cut-off value of 3.6 Å is considered for N⋯O non-bonding distances and *A*—O bond lengths. The calculated values (Brese & O’Keeffe 1991 ▸) of bond-valence sums for W^6+^, Se^4+^ and monovalent alkali metal ions are in the 6.079–6.283, 3.807–3.975 and 0.060–1.275 ranges, respectively. The respective values of 3.210, 3.322 and 3.207 Å for the shortest inter­layer O⋯O non-bonding distances of compounds **1**–**3** with intra-layer Se—O bonds are significantly higher than the corresponding value of 2.563 Å for compound **4α** with inter­layer Se—O bonds.

The net dipole moment values for the WO_6_ and SeO_3_ polyhedra were calculated by vector summation of the dipole moments (Maggard *et al.*, 2003 ▸; Ok & Halasyamani 2005 ▸; Galy *et al.*, 1975 ▸) of six W—O bonds and three Se—O bonds and found to be in the 0.79–1.85 D and 5.73–9.13 D ranges, respectively (Tables S1–S3). The net dipole for the WO_6_ octa­hedron points towards the triangular face of three oxygen atoms with W—O bonds >1.9 Å long, whereas the net dipoles for the SeO_3_ polyhedra point opposite to the lone pair of electrons of selenium. In compounds **1**–**3**, as shown for Rb_2_W_3_SeO_12_ (**3**) in Fig. 1 ▸, the intra-layer SeO_3_ dipole is oriented along the *c*-axis direction and perpendicular to the HTO layer. For the WO_6_ octa­hedra, the net dipole moment components along the *a* and *b* axes cancel one another, whereas the *c*-axis component is anti­parallel and additive to the net dipole moment of pyramidal SeO_3_. In the case of centrosymmetric three-dimensional K_2_W_3_SeO_12_ (**4α**), as shown in Fig. 2 ▸, the net dipole moments of the WO_6_ and SeO_3_ polyhedra macroscopically cancel one another and result in a zero net dipole moment.

**Figure 3:**
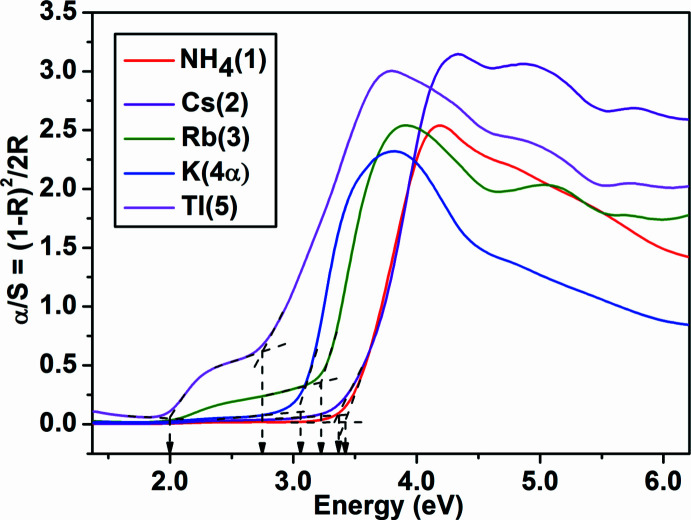
Solid state UV–visible absorption spectra for the *A*_2_W_3_SeO_12_ [*A* = NH_4_ (**1**), Cs (**2**), Rb (**3**), K (**4α**) and Tl (**5**)] compounds.

The solid state UV–Visible absorption spectra (Fig. 3 ▸) of compounds **1**–**5** reveal that their band gap values are in the range 2.7–3.5 eV (Kubelka & Munk, 1931 ▸). The additional absorption edge observed for the Tl_2_W_3_SeO_12_ compound (**5**) corresponds to band gap value of 2.0 eV. When compared to Cs_2_W_3_SeO_12_ (**2**), the corresponding Cs_2_W_3_TeO_12_ tellurite (Zhao *et al.*, 2015 ▸) has a lower band gap of 2.89 eV.

Rb_2_W_3_SeO_12_ (**3**), K_2_W_3_SeO_12_ (**4α**) and Tl_2_W_3_SeO_12_ (**5**) undergo thermal decompositions and give rise to endothermic peaks at ∼600, ∼575 and ∼575°C and their respective observed weight losses of 10.0%, 12.3% and 9.0% compare well with those calculated for the loss of SeO_2_ (Figure S3). The other endothermic peaks at ∼850 and 750°C could not be assigned. It was reported (Harrison *et al.*, 1995 ▸) that a similar thermal loss of SeO_2_ occurs in a single step between 500 and 600°C for Cs_2_W_3_SeO_12_ (**2**) and in two steps at 350 and 450°C for (NH_4_)_2_W_3_SeO_12_ (**1**). When compared to the tungsten selenites **1**–**5**, analogous *A*_2_W_3_TeO_12_ (*A* = K, Rb, Cs) tellurites of tungsten (Goodey *et al.*, 2003 ▸; Zhao *et al.*, 2015 ▸) and *A*_2_Mo_3_SeO_12_ (*A* = Rb, Tl) selenites of molybdenum (Chang *et al.*, 2010 ▸) undergo single-step thermal decomposition at higher and lower temperatures of >700 and 300°C, respectively.

## Syntheses and crystallization   

Cs_2_CO_3_ (Alfa Aesar), Rb_2_CO_3_ (Alfa Aesar), TlNO_3_ (Sigma Aldrich), H_2_WO_4_ (Sigma Aldrich), SeO_2_ (Sigma Aldrich), NH_4_Cl (Sarabhai M Chemicals) of >99% purity, NH_4_OH (Fischer Scientific) of 25% dilution, WO_3_ and Tl_2_WO_4_ were used for the synthesis and crystal growth of compounds **1**–**5**. WO_3_ was obtained by heating H_2_WO_4_ in the open air. Tl_2_WO_4_ was prepared by heating a stoichiometric mixture of TlNO_3_ and H_2_WO_4_. Teflon-lined stainless steel acid digestion vessels of 23 mL capacity were employed for the hydro­thermal reactions.

The reactants and their qu­anti­ties, the temperature and duration of heating and the yields of products for the synthesis and crystal growth of compounds **1**–**5** are presented in Table S4. The ammonium compound (**1**) was synthesized by the hydro­thermal method, with or without NH_4_Cl as mineralizer. The other four compounds (**2**–**5)** were obtained by solid-state reactions. The reactant mixtures were heated first in the open air and later in evacuated sealed silica ampoules. After the reaction, the solid product contents were washed with water to dissolve away the excess SeO_2_.

The hydro­thermal and solid-state synthetic methods enabled the growth and isolation of single crystals of compounds **1**–**5.** The utilization of excess SeO_2_ as flux in the novel solid-state synthetic procedure facilitated the growth of single crystals of compounds **2**–**5**. The powder XRD patterns of compounds **1**–**5** are presented in Figures S1a and S1b. (NH_4_)_2_W_3_SeO_12_ (**1**), Rb_2_W_3_SeO_12_ (**3**) and Tl_2_W_3_SeO_12_ (**5**) were obtained as homogeneous phases, as their observed powder XRD patterns compare reasonably well with the simulated ones. The powder XRD patterns of Cs_2_W_3_SeO_12_ (**2**), Rb_2_W_3_SeO_12_ (**3**) and K_2_W_3_SeO_12_ (**4α**) contained two or three additional reflections of <10% intensity due to WO_3_ or an unidentified phase; however, the homogeneous polycrystalline sample of Rb_2_W_3_SeO_12_ (**3**) could be obtained (Figure S1b), under a different set of solid-state synthetic conditions mentioned in Table S4. Cs_2_W_3_SeO_12_ (**2**) was prepared in polycrystalline form by the reported hydro­thermal method (Harrison *et al.*, 1995 ▸). It is evident from the scanning electron micrographs (Figure S4) that crystallites of compounds **1** and **2** have a hexa­gonal prism shape and compounds **3**–**5** have block-shaped morphologies. The EDXA analyses confirmed the expected ratios of metal contents for all compounds **1**–**5**.

## Refinement   

Crystal data, data collection and structure refinement details are summarized in Table 1 ▸. The crystals of the ammonium (**1**) and rubidium (**3**) compounds are twinned by merohedry (Spek 2020 ▸) by the [−1 0 0 1 1 0 0 0 − 1] and [1 0 0 − 1 −1 0 0 0 − 1] twin laws and their twinned lattices are generated through twofold rotation of the primary lattices about the [120] direction and the *b* axis, respectively. The crystal of the potassium (**4α**) compound is twinned by pseudo-merohedry (Spek 2020 ▸) by the twin law [−1 0 0 0 − 1 0 0 0 1] and the twinned lattice is generated through twofold rotation of the primary lattice about the ***c*** axis, as the value of the β angle of its monoclinic system is very close to 90°. The respective values of refined batch scale factor for the ammonium (**1**), rubidium (**3**) and potassium (**4α**) compounds are 0.029, 0.192 and 0.385. The hydrogen atoms of the NH_4_^+^ ions in the ammonium compound (**1**) were not located in the difference-Fourier maps but are included in the formula. The final difference-Fourier maps did not show any chemically significant features and the Fourier difference peaks with an electron density of >1 e Å^−3^ were found to be ghosts. No reasonable structure solutions and refinements in the centrosymmetric *P*6_3_/*m* space group were found for compounds **1**–**3**.

The powder X-ray diffraction (XRD) patterns of compounds **1**–**5** were recorded on a Bruker D8 Advanced powder X-ray diffractometer using Cu *K*α (*λ =* 1.5418 Å) radiation. The monophasic nature of each of these compounds was verified by comparing their powder XRD patterns with those simulated, using *Mercury* (Macrae *et al.*, 2020 ▸), on the basis of their single crystal X-ray structures.

## Supplementary Material

Crystal structure: contains datablock(s) global, NH42W3SeO121, Cs2W3SeO122, Rb2W3SeO123, K2W3SeO124. DOI: 10.1107/S2056989021002735/ru2074sup1.cif

Structure factors: contains datablock(s) NH42W3SeO121. DOI: 10.1107/S2056989021002735/ru2074NH42W3SeO121sup2.hkl

Supporting information file. DOI: 10.1107/S2056989021002735/ru2074sup6.pdf

Rietveld powder data: contains datablock(s) NH42W3SeO121. DOI: 10.1107/S2056989021002735/ru2074NH42W3SeO121sup7.rtv

Structure factors: contains datablock(s) Cs2W3SeO122. DOI: 10.1107/S2056989021002735/ru2074Cs2W3SeO122sup3.hkl

Rietveld powder data: contains datablock(s) Cs2W3SeO122. DOI: 10.1107/S2056989021002735/ru2074Cs2W3SeO122sup8.rtv

Structure factors: contains datablock(s) Rb2W3SeO123. DOI: 10.1107/S2056989021002735/ru2074Rb2W3SeO123sup4.hkl

Rietveld powder data: contains datablock(s) Rb2W3SeO123. DOI: 10.1107/S2056989021002735/ru2074Rb2W3SeO123sup9.rtv

Rietveld powder data: contains datablock(s) K2W3SeO124. DOI: 10.1107/S2056989021002735/ru2074K2W3SeO124sup10.rtv

Structure factors: contains datablock(s) K2W3SeO124. DOI: 10.1107/S2056989021002735/ru2074K2W3SeO124sup5.hkl

CCDC references: 2000910, 2000899, 2000898, 2000897

Additional supporting information:  crystallographic information; 3D view; checkCIF report

## Figures and Tables

**Table 1:** Experimental details

	**1**	**2**	**3**	**4**
Crystal data				
Chemical formula	(NH_4_)_2_W_3_SeO_12_	Cs_2_W_3_SeO_12_	Rb_2_W_3_SeO_12_	K_2_W_3_SeO_12_
*M*_r_	858.59	1088.33	993.40	900.66
Crystal system, space group	Hexagonal, *P*6_3_	Hexagonal, *P*6_3_	Hexagonal, *P*6_3_	Monoclinic, *P*2_1_/*n*
Temperature (K)	297	293	293	293
*a*, *b*, *c* (Å)	7.2303 (3), 7.2303 (3), 12.1491 (5)	7.2580 (3), 7.2580 (3), 12.5291 (5)	7.2380 (1), 7.2380 (1), 12.1115 (3)	7.2310 (2), 11.4863 (4), 12.6486 (4)
α, β, γ (°)	90, 90, 120	90, 90, 120	90, 90, 120	90, 90.096 (2), 90
*V* (Å^3^)	550.03 (5)	571.59 (5)	549.50 (2)	1050.56 (6)
*Z*	2	2	2	4
Radiation type	Mo *K*α	Mo *K*α	Mo *K*α	Mo *K*α
μ (mm^−1^)	34.67	39.63	43.49	37.09
Crystal size (mm)	0.10 × 0.10 × 0.05	0.10 × 0.08 × 0.05	0.10 × 0.05 × 0.05	0.08 × 0.05 × 0.02

Data collection				
Diffractometer	Bruker APEXII CCD	Bruker APEXII CCD	Bruker APEXII CCD	Bruker APEXII CCD
Absorption correction	Multi-scan (*SADABS*; Krause *et al.*, 2015 ▸)	Multi-scan (*SADABS*; Krause *et al.*, 2015 ▸)	Multi-scan (*SADABS*; Krause *et al.*, 2015 ▸)	Multi-scan (*SADABS*; Krause *et al.*, 2015 ▸)
*T*_min_, *T*_max_	0.129, 0.276	0.110, 0.242	0.098, 0.220	0.155, 0.524
No. of measured, independent and observed [*I* > 2σ(*I*)] reflections	14419, 1079, 1069	2825, 831, 730	3497, 675, 654	26562, 4026, 3456
*R*_int_	0.049	0.058	0.033	0.073
(sin θ/λ)_max_ (Å^−1^)	0.702	0.643	0.666	0.770

Refinement				
*R*[*F* ^2^ > 2σ(*F* ^2^)], *wR*(*F* ^2^), *S*	0.023, 0.052, 1.27	0.029, 0.054, 1.02	0.018, 0.033, 1.06	0.032, 0.105, 1.15
No. of reflections	1079	831	675	4026
No. of parameters	57	56	57	165
No. of restraints	1	13	7	24
H-atom treatment	H-atom parameters not defined	–	–	–
Δρ_max_, Δρ_min_ (e Å^−3^)	2.63, −2.37	1.90, −1.65	0.90, −1.14	3.94, −3.11
Absolute structure	Flack *x* determined using 492 quotients [(*I* ^+^)−(*I* ^−^)]/[(*I* ^+^)+(*I* ^−^)] (Parsons *et al.*, 2013 ▸)	Flack *x* determined using 297 quotients [(*I* ^+^)−(*I* ^−^)]/[(*I* ^+^)+(*I* ^−^)] (Parsons *et al.*, 2013 ▸)	Flack *x* determined using 182 quotients [(*I* ^+^)−(*I* ^−^)]/[(*I* ^+^)+(*I* ^−^)] (Parsons *et al.*, 2013 ▸)	
Absolute structure parameter	0.015 (13)	0.00 (3)	−0.08 (3)	–

Computer programs: *APEX2* and *SAINT* (Bruker, 2004 ▸), *SHELXT* (Sheldrick, 2015*a* ▸), *SHELXL2013* (Sheldrick, 2015*b* ▸), *SHELXL* (Sheldrick, 2015*b* ▸), *ORTEP-3 for Windows* (Farrugia, 2012 ▸), *DIAMOND* (Pennington, 1999 ▸) and *SHELXL97* (Sheldrick, 2008 ▸).

## supplementary crystallographic information

### Diammonium tritungsten selenite (NH42W3SeO121) Crystal data

**Table:**

(NH_4_)_2_W_3_SeO_12_	*D*_x_ = 5.184 Mg m^−^^3^
*M**_r_* = 858.59	Mo *K*α radiation, λ = 0.71073 Å
Hexagonal, *P*6_3_	Cell parameters from 7856 reflections
*a* = 7.2303 (3) Å	θ = 3.3–30.4°
*c* = 12.1491 (5) Å	µ = 34.67 mm^−^^1^
*V* = 550.03 (5) Å^3^	*T* = 297 K
*Z* = 2	Block, colourless
*F*(000) = 748	0.10 × 0.10 × 0.05 mm

### Diammonium tritungsten selenite (NH42W3SeO121) Data collection

**Table:**

Bruker APEXII CCD diffractometer	1069 reflections with *I* > 2σ(*I*)
Radiation source: fine-focus sealed tube	*R*_int_ = 0.049
φ and ω scans	θ_max_ = 30.0°, θ_min_ = 3.4°
Absorption correction: multi-scan (SADABS; Krause *et al.*, 2015)	*h* = −10→10
*T*_min_ = 0.129, *T*_max_ = 0.276	*k* = −10→10
14419 measured reflections	*l* = −17→17
1079 independent reflections	

### Diammonium tritungsten selenite (NH42W3SeO121) Refinement

**Table:**

Refinement on *F*^2^	*w* = 1/[σ^2^(*F*_o_^2^) + (0.0097*P*)^2^ + 8.3615*P*] where *P* = (*F*_o_^2^ + 2*F*_c_^2^)/3
Least-squares matrix: full	(Δ/σ)_max_ < 0.001
*R*[*F*^2^ > 2σ(*F*^2^)] = 0.023	Δρ_max_ = 2.63 e Å^−^^3^
*wR*(*F*^2^) = 0.052	Δρ_min_ = −2.37 e Å^−^^3^
*S* = 1.27	Extinction correction: SHELXL-2018/3 (Sheldrick 2015b), Fc^*^=kFc[1+0.001xFc^2^λ^3^/sin(2θ)]^-1/4^
1079 reflections	Extinction coefficient: 0.0100 (5)
57 parameters	Absolute structure: Flack *x* determined using 492 quotients [(*I*^+^)-(*I*^-^)]/[(*I*^+^)+(*I*^-^)] (Parsons *et al.*, 2013)
1 restraint	Absolute structure parameter: 0.015 (13)
H-atom parameters not defined	

### Diammonium tritungsten selenite (NH42W3SeO121) Special details

**Table:**

Experimental. Single crystals of compounds 1–5 were obtained along with the polycrystalline sample and the crystals were hand-picked for XRD study and mounted on thin glass fibres with epoxy glue and optically aligned on a Bruker APEXII charge-coupled device X-ray diffractometer using a digital camera. Intensity data were measured at 25 ^°^C using Mo Kα (λ = 0.7103 Å) radiation. APEX II software (Bruker AXS) was used for preliminary determination of the cell constants and data collection control. The determination of integral intensities and global refinement were performed using SAINT-plus (Bruker AXS). A semi-empirical absorption correction was subsequently applied using SADABS. Space group determination, structure solution and least-squares refinement were carried out using SHELXTL (Sheldrick 2008) program. DIAMOND 3.0 (PENNINGTON 1999) and ORTEP-3 (Farrugia 1997) for windows were the graphic programs employed to draw the structures. The structures were solved by direct methods and refined by full matrix least squares on F^2^.
Geometry. All esds (except the esd in the dihedral angle between two l.s. planes) are estimated using the full covariance matrix. The cell esds are taken into account individually in the estimation of esds in distances, angles and torsion angles; correlations between esds in cell parameters are only used when they are defined by crystal symmetry. An approximate (isotropic) treatment of cell esds is used for estimating esds involving l.s. planes.
Refinement. Refined as a two-component twin

### Diammonium tritungsten selenite (NH42W3SeO121) Fractional atomic coordinates and isotropic or equivalent isotropic displacement parameters (Å^2^)

**Table:**

	*x*	*y*	*z*	*U*_iso_*/*U*_eq_	
N1	0.333333	0.666667	0.115 (3)	0.042 (8)	
N2	0.666667	0.333333	0.237 (2)	0.025 (5)	
W	0.19142 (6)	0.33979 (5)	0.40000 (10)	0.00766 (15)	
Se	0.000000	0.000000	0.16680 (15)	0.0102 (5)	
O1	0.1264 (15)	0.2465 (16)	0.2271 (7)	0.0134 (17)	
O2	0.4083 (16)	0.1964 (16)	0.0370 (8)	0.0137 (18)	
O3	0.2494 (12)	0.1188 (12)	0.4103 (12)	0.0101 (19)	
O4	0.0850 (15)	0.5365 (14)	0.3526 (8)	0.0111 (16)	

### Diammonium tritungsten selenite (NH42W3SeO121) Atomic displacement parameters (Å^2^)

**Table:**

	*U*^11^	*U*^22^	*U*^33^	*U*^12^	*U*^13^	*U*^23^
N1	0.052 (13)	0.052 (13)	0.023 (14)	0.026 (7)	0.000	0.000
N2	0.025 (8)	0.025 (8)	0.024 (12)	0.012 (4)	0.000	0.000
W	0.0069 (2)	0.00476 (19)	0.0108 (2)	0.00250 (14)	−0.0007 (3)	−0.0006 (3)
Se	0.0091 (5)	0.0091 (5)	0.0124 (12)	0.0045 (2)	0.000	0.000
O1	0.013 (4)	0.010 (4)	0.015 (4)	0.005 (4)	−0.004 (3)	0.001 (3)
O2	0.010 (4)	0.013 (5)	0.016 (4)	0.004 (4)	−0.001 (3)	0.002 (3)
O3	0.006 (3)	0.006 (3)	0.019 (5)	0.003 (2)	−0.002 (5)	0.005 (5)
O4	0.011 (4)	0.004 (4)	0.018 (4)	0.004 (3)	−0.003 (3)	−0.002 (3)

### Diammonium tritungsten selenite (NH42W3SeO121) Geometric parameters (Å, º)

**Table:**

W—O2^i^	1.722 (10)	W—O1	2.184 (9)
W—O3	1.848 (8)	Se—O1^iii^	1.709 (10)
W—O4^ii^	1.874 (9)	Se—O1	1.709 (10)
W—O3^iii^	1.985 (8)	Se—O1^iv^	1.709 (10)
W—O4	2.010 (9)		
			
O2^i^—W—O3	99.2 (6)	O3—W—O1	84.5 (5)
O2^i^—W—O4^ii^	99.3 (5)	O4^ii^—W—O1	86.0 (4)
O3—W—O4^ii^	96.7 (4)	O3^iii^—W—O1	80.7 (5)
O2^i^—W—O3^iii^	93.4 (6)	O4—W—O1	81.0 (4)
O3—W—O3^iii^	89.7 (4)	O1^iii^—Se—O1	102.9 (4)
O4^ii^—W—O3^iii^	164.6 (5)	O1^iii^—Se—O1^iv^	102.9 (4)
O2^i^—W—O4	94.7 (4)	O1—Se—O1^iv^	102.9 (4)
O3—W—O4	164.5 (5)	Se—O1—W	130.9 (5)
O4^ii^—W—O4	87.8 (6)	W—O3—W^iv^	149.1 (5)
O3^iii^—W—O4	82.6 (4)	W^v^—O4—W	132.5 (5)
O2^i^—W—O1	173.1 (4)		

Symmetry codes: (i) *x*−*y*, *x*, *z*+1/2; (ii) −*y*+1, *x*−*y*+1, *z*; (iii) −*y*, *x*−*y*, *z*; (iv) −*x*+*y*, −*x*, *z*; (v) −*x*+*y*, −*x*+1, *z*.

### Dicaesium tritungsten selenite (Cs2W3SeO122) Crystal data

**Table:**

Cs_2_W_3_SeO_12_	*D*_x_ = 6.323 Mg m^−^^3^
*M**_r_* = 1088.33	Mo *K*α radiation, λ = 0.71073 Å
Hexagonal, *P*6_3_	Cell parameters from 706 reflections
*a* = 7.2580 (3) Å	θ = 3.6–26.3°
*c* = 12.5291 (5) Å	µ = 39.63 mm^−^^1^
*V* = 571.59 (5) Å^3^	*T* = 293 K
*Z* = 2	Block, colourless
*F*(000) = 924	0.10 × 0.08 × 0.05 mm

### Dicaesium tritungsten selenite (Cs2W3SeO122) Data collection

**Table:**

Bruker APEXII CCD diffractometer	730 reflections with *I* > 2σ(*I*)
Radiation source: fine-focus sealed tube	*R*_int_ = 0.058
phi and ω scans	θ_max_ = 27.2°, θ_min_ = 3.2°
Absorption correction: multi-scan (SADABS; Krause *et al.*, 2015)	*h* = −6→8
*T*_min_ = 0.110, *T*_max_ = 0.242	*k* = −9→9
2825 measured reflections	*l* = −16→14
831 independent reflections	

### Dicaesium tritungsten selenite (Cs2W3SeO122) Refinement

**Table:**

Refinement on *F*^2^	*w* = 1/[σ^2^(*F*_o_^2^)] where *P* = (*F*_o_^2^ + 2*F*_c_^2^)/3
Least-squares matrix: full	(Δ/σ)_max_ < 0.001
*R*[*F*^2^ > 2σ(*F*^2^)] = 0.029	Δρ_max_ = 1.90 e Å^−^^3^
*wR*(*F*^2^) = 0.054	Δρ_min_ = −1.65 e Å^−^^3^
*S* = 1.02	Extinction correction: SHELXL2018/3 (Sheldrick 2015b), Fc^*^=kFc[1+0.001xFc^2^λ^3^/sin(2θ)]^-1/4^
831 reflections	Extinction coefficient: 0.0081 (4)
56 parameters	Absolute structure: Flack *x* determined using 297 quotients [(*I*^+^)-(*I*^-^)]/[(*I*^+^)+(*I*^-^)] (Parsons *et al.*, 2013)
13 restraints	Absolute structure parameter: 0.00 (3)

### Dicaesium tritungsten selenite (Cs2W3SeO122) Special details

**Table:**

Geometry. All esds (except the esd in the dihedral angle between two l.s. planes) are estimated using the full covariance matrix. The cell esds are taken into account individually in the estimation of esds in distances, angles and torsion angles; correlations between esds in cell parameters are only used when they are defined by crystal symmetry. An approximate (isotropic) treatment of cell esds is used for estimating esds involving l.s. planes.

### Dicaesium tritungsten selenite (Cs2W3SeO122) Fractional atomic coordinates and isotropic or equivalent isotropic displacement parameters (Å^2^)

**Table:**

	*x*	*y*	*z*	*U*_iso_*/*U*_eq_	
Cs1	0.333333	0.666667	0.0902 (2)	0.0269 (9)	
Cs2	0.666667	0.333333	0.22953 (19)	0.0143 (7)	
W	0.14949 (10)	0.33884 (11)	0.39443 (8)	0.0056 (2)	
Se	0.000000	0.000000	0.1700 (2)	0.0058 (7)	
O1	0.125 (2)	0.250 (2)	0.2270 (11)	0.007 (3)	
O2	0.401 (2)	0.207 (2)	0.0265 (14)	0.015 (3)	
O3	0.2495 (18)	0.1279 (17)	0.4056 (14)	0.008 (3)	
O4	0.088 (2)	0.549 (2)	0.3529 (11)	0.010 (3)	

### Dicaesium tritungsten selenite (Cs2W3SeO122) Atomic displacement parameters (Å^2^)

**Table:**

	*U*^11^	*U*^22^	*U*^33^	*U*^12^	*U*^13^	*U*^23^
Cs1	0.0277 (13)	0.0277 (13)	0.0251 (16)	0.0139 (6)	0.000	0.000
Cs2	0.0160 (10)	0.0160 (10)	0.0110 (13)	0.0080 (5)	0.000	0.000
W	0.0048 (4)	0.0031 (4)	0.0083 (4)	0.0016 (3)	0.0004 (5)	−0.0001 (5)
Se	0.0066 (9)	0.0066 (9)	0.0042 (18)	0.0033 (5)	0.000	0.000
O1	0.013 (8)	0.004 (7)	0.002 (6)	0.004 (7)	0.000 (6)	−0.001 (6)
O2	0.015 (8)	0.011 (9)	0.020 (9)	0.007 (7)	−0.010 (7)	0.003 (7)
O3	0.008 (3)	0.008 (3)	0.009 (3)	0.0042 (18)	0.0001 (14)	−0.0003 (14)
O4	0.009 (3)	0.009 (3)	0.010 (3)	0.0050 (19)	0.0000 (14)	−0.0001 (14)

### Dicaesium tritungsten selenite (Cs2W3SeO122) Geometric parameters (Å, º)

**Table:**

Cs1—O1^i^	3.132 (15)	Cs2—O4^ii^	3.066 (14)
Cs1—O1	3.132 (15)	Cs2—O3^vi^	3.427 (13)
Cs1—O1^ii^	3.132 (15)	Cs2—O3	3.427 (13)
Cs1—O3^iii^	3.497 (15)	Cs2—O3^vii^	3.427 (13)
Cs1—O3^iv^	3.497 (15)	Cs2—O1^vii^	3.667 (13)
Cs1—O3^v^	3.497 (15)	Cs2—O1	3.667 (13)
Cs1—O4^i^	3.634 (14)	Cs2—O1^vi^	3.667 (13)
Cs1—O4^ii^	3.634 (14)	W—O2^x^	1.703 (17)
Cs1—O4	3.634 (14)	W—O3^xi^	1.840 (11)
Cs1—O2^i^	3.695 (14)	W—O4	1.863 (13)
Cs1—O2^ii^	3.695 (14)	W—O4^ii^	2.000 (13)
Cs1—O2	3.695 (14)	W—O3	2.000 (10)
Cs2—O2	3.044 (16)	W—O1	2.176 (14)
Cs2—O2^vi^	3.044 (16)	Se—O1^xi^	1.723 (15)
Cs2—O2^vii^	3.044 (16)	Se—O1	1.724 (15)
Cs2—O4^viii^	3.066 (14)	Se—O1^ix^	1.724 (15)
Cs2—O4^ix^	3.066 (14)		
			
O1^i^—Cs1—O1	92.9 (4)	O4^ii^—Cs2—O1^vi^	143.4 (3)
O1^i^—Cs1—O1^ii^	92.9 (4)	O3^vi^—Cs2—O1^vi^	45.0 (3)
O1—Cs1—O1^ii^	92.9 (4)	O3—Cs2—O1^vi^	97.0 (3)
O1^i^—Cs1—O3^iii^	74.6 (3)	O3^vii^—Cs2—O1^vi^	127.0 (3)
O1—Cs1—O3^iii^	132.9 (3)	O1^vii^—Cs2—O1^vi^	119.992 (7)
O1^ii^—Cs1—O3^iii^	132.1 (3)	O1—Cs2—O1^vi^	119.993 (8)
O1^i^—Cs1—O3^iv^	132.1 (3)	O2^x^—W—O3^xi^	97.7 (8)
O1—Cs1—O3^iv^	74.6 (3)	O2^x^—W—O4	98.5 (7)
O1^ii^—Cs1—O3^iv^	132.9 (3)	O3^xi^—W—O4	96.6 (5)
O3^iii^—Cs1—O3^iv^	81.0 (4)	O2^x^—W—O4^ii^	93.8 (6)
O1^i^—Cs1—O3^v^	132.9 (3)	O3^xi^—W—O4^ii^	167.0 (7)
O1—Cs1—O3^v^	132.1 (3)	O4—W—O4^ii^	87.4 (8)
O1^ii^—Cs1—O3^v^	74.6 (3)	O2^x^—W—O3	92.3 (7)
O3^iii^—Cs1—O3^v^	81.0 (4)	O3^xi^—W—O3	89.9 (7)
O3^iv^—Cs1—O3^v^	81.0 (4)	O4—W—O3	166.5 (7)
O1^i^—Cs1—O4^i^	48.2 (3)	O4^ii^—W—O3	83.7 (5)
O1—Cs1—O4^i^	81.9 (3)	O2^x^—W—O1	172.7 (6)
O1^ii^—Cs1—O4^i^	47.2 (3)	O3^xi^—W—O1	85.8 (7)
O3^iii^—Cs1—O4^i^	116.3 (3)	O4—W—O1	87.5 (6)
O3^iv^—Cs1—O4^i^	156.5 (3)	O4^ii^—W—O1	82.1 (5)
O3^v^—Cs1—O4^i^	115.9 (3)	O3—W—O1	81.2 (6)
O1^i^—Cs1—O4^ii^	81.9 (3)	O2^x^—W—Cs2^xii^	123.2 (5)
O1—Cs1—O4^ii^	47.2 (3)	O3^xi^—W—Cs2^xii^	57.3 (4)
O1^ii^—Cs1—O4^ii^	48.2 (3)	O4—W—Cs2^xii^	46.0 (4)
O3^iii^—Cs1—O4^ii^	156.5 (3)	O4^ii^—W—Cs2^xii^	120.1 (4)
O3^iv^—Cs1—O4^ii^	115.9 (3)	O3—W—Cs2^xii^	132.2 (4)
O3^v^—Cs1—O4^ii^	116.3 (3)	O1—W—Cs2^xii^	64.1 (3)
O4^i^—Cs1—O4^ii^	43.1 (4)	O2^x^—W—Cs1^xiii^	59.3 (5)
O1^i^—Cs1—O4	47.2 (3)	O3^xi^—W—Cs1^xiii^	53.6 (5)
O1—Cs1—O4	48.2 (3)	O4—W—Cs1^xiii^	68.7 (4)
O1^ii^—Cs1—O4	81.9 (3)	O4^ii^—W—Cs1^xiii^	138.9 (4)
O3^iii^—Cs1—O4	115.9 (3)	O3—W—Cs1^xiii^	124.3 (4)
O3^iv^—Cs1—O4	116.3 (3)	O1—W—Cs1^xiii^	127.4 (3)
O3^v^—Cs1—O4	156.5 (3)	Cs2^xii^—W—Cs1^xiii^	65.81 (5)
O4^i^—Cs1—O4	43.1 (4)	O2^x^—W—Cs2	114.7 (5)
O4^ii^—Cs1—O4	43.1 (4)	O3^xi^—W—Cs2	127.9 (4)
O1^i^—Cs1—O2^i^	57.5 (4)	O4—W—Cs2	116.1 (4)
O1—Cs1—O2^i^	91.3 (3)	O4^ii^—W—Cs2	40.4 (4)
O1^ii^—Cs1—O2^i^	150.4 (4)	O3—W—Cs2	51.3 (4)
O3^iii^—Cs1—O2^i^	43.5 (3)	O1—W—Cs2	58.5 (3)
O3^iv^—Cs1—O2^i^	76.4 (3)	Cs2^xii^—W—Cs2	120.63 (6)
O3^v^—Cs1—O2^i^	122.2 (4)	Cs1^xiii^—W—Cs2	173.49 (5)
O4^i^—Cs1—O2^i^	104.7 (4)	O2^x^—W—Cs1	138.0 (6)
O4^ii^—Cs1—O2^i^	121.5 (3)	O3^xi^—W—Cs1	116.2 (5)
O4—Cs1—O2^i^	79.1 (3)	O4—W—Cs1	55.9 (4)
O1^i^—Cs1—O2^ii^	91.3 (3)	O4^ii^—W—Cs1	56.4 (4)
O1—Cs1—O2^ii^	150.4 (4)	O3—W—Cs1	110.6 (5)
O1^ii^—Cs1—O2^ii^	57.5 (4)	O1—W—Cs1	43.4 (4)
O3^iii^—Cs1—O2^ii^	76.4 (3)	Cs2^xii^—W—Cs1	65.44 (3)
O3^iv^—Cs1—O2^ii^	122.2 (4)	Cs1^xiii^—W—Cs1	122.50 (3)
O3^v^—Cs1—O2^ii^	43.5 (3)	Cs2—W—Cs1	63.41 (3)
O4^i^—Cs1—O2^ii^	79.1 (3)	O2^x^—W—Cs1^xiv^	54.8 (5)
O4^ii^—Cs1—O2^ii^	104.7 (4)	O3^xi^—W—Cs1^xiv^	120.7 (4)
O4—Cs1—O2^ii^	121.5 (3)	O4—W—Cs1^xiv^	134.5 (4)
O2^i^—Cs1—O2^ii^	115.47 (19)	O4^ii^—W—Cs1^xiv^	62.2 (4)
O1^i^—Cs1—O2	150.4 (4)	O3—W—Cs1^xiv^	48.1 (4)
O1—Cs1—O2	57.5 (4)	O1—W—Cs1^xiv^	117.9 (3)
O1^ii^—Cs1—O2	91.3 (3)	Cs2^xii^—W—Cs1^xiv^	177.46 (4)
O3^iii^—Cs1—O2	122.2 (4)	Cs1^xiii^—W—Cs1^xiv^	111.82 (6)
O3^iv^—Cs1—O2	43.5 (3)	Cs2—W—Cs1^xiv^	61.72 (4)
O3^v^—Cs1—O2	76.4 (3)	Cs1—W—Cs1^xiv^	117.06 (2)
O4^i^—Cs1—O2	121.5 (3)	O1^xi^—Se—O1	104.0 (5)
O4^ii^—Cs1—O2	79.1 (3)	O1^xi^—Se—O1^ix^	104.0 (5)
O4—Cs1—O2	104.7 (4)	O1—Se—O1^ix^	104.0 (5)
O2^i^—Cs1—O2	115.47 (19)	O1^xi^—Se—Cs2^xv^	58.6 (4)
O2^ii^—Cs1—O2	115.47 (19)	O1—Se—Cs2^xv^	145.4 (5)
O2—Cs2—O2^vi^	56.8 (5)	O1^ix^—Se—Cs2^xv^	58.6 (4)
O2—Cs2—O2^vii^	56.8 (5)	O1^xi^—Se—Cs2	145.4 (5)
O2^vi^—Cs2—O2^vii^	56.8 (5)	O1—Se—Cs2	58.6 (4)
O2—Cs2—O4^viii^	153.5 (4)	O1^ix^—Se—Cs2	58.6 (4)
O2^vi^—Cs2—O4^viii^	101.6 (4)	Cs2^xv^—Se—Cs2	116.99 (3)
O2^vii^—Cs2—O4^viii^	99.6 (4)	O1^xi^—Se—Cs2^xii^	58.6 (4)
O2—Cs2—O4^ix^	101.6 (4)	O1—Se—Cs2^xii^	58.6 (4)
O2^vi^—Cs2—O4^ix^	99.6 (4)	O1^ix^—Se—Cs2^xii^	145.4 (5)
O2^vii^—Cs2—O4^ix^	153.5 (4)	Cs2^xv^—Se—Cs2^xii^	116.99 (3)
O4^viii^—Cs2—O4^ix^	96.8 (3)	Cs2—Se—Cs2^xii^	116.99 (3)
O2—Cs2—O4^ii^	99.6 (4)	O1^xi^—Se—Cs1^xv^	37.9 (5)
O2^vi^—Cs2—O4^ii^	153.5 (4)	O1—Se—Cs1^xv^	122.6 (4)
O2^vii^—Cs2—O4^ii^	101.6 (4)	O1^ix^—Se—Cs1^xv^	122.6 (4)
O4^viii^—Cs2—O4^ii^	96.8 (3)	Cs2^xv^—Se—Cs1^xv^	64.012 (17)
O4^ix^—Cs2—O4^ii^	96.8 (3)	Cs2—Se—Cs1^xv^	176.68 (9)
O2—Cs2—O3^vi^	139.0 (3)	Cs2^xii^—Se—Cs1^xv^	64.014 (17)
O2^vi^—Cs2—O3^vi^	96.8 (4)	O1^xi^—Se—Cs1	122.6 (4)
O2^vii^—Cs2—O3^vi^	137.8 (3)	O1—Se—Cs1	37.9 (5)
O4^viii^—Cs2—O3^vi^	50.0 (3)	O1^ix^—Se—Cs1	122.6 (4)
O4^ix^—Cs2—O3^vi^	48.2 (3)	Cs2^xv^—Se—Cs1	176.68 (9)
O4^ii^—Cs2—O3^vi^	109.7 (4)	Cs2—Se—Cs1	64.013 (17)
O2—Cs2—O3	96.8 (4)	Cs2^xii^—Se—Cs1	64.013 (17)
O2^vi^—Cs2—O3	137.8 (3)	Cs1^xv^—Se—Cs1	114.79 (4)
O2^vii^—Cs2—O3	139.0 (3)	O1^xi^—Se—Cs1^xvi^	122.6 (4)
O4^viii^—Cs2—O3	109.7 (4)	O1—Se—Cs1^xvi^	122.6 (4)
O4^ix^—Cs2—O3	50.0 (3)	O1^ix^—Se—Cs1^xvi^	37.9 (5)
O4^ii^—Cs2—O3	48.2 (3)	Cs2^xv^—Se—Cs1^xvi^	64.014 (17)
O3^vi^—Cs2—O3	83.0 (4)	Cs2—Se—Cs1^xvi^	64.013 (17)
O2—Cs2—O3^vii^	137.8 (3)	Cs2^xii^—Se—Cs1^xvi^	176.68 (9)
O2^vi^—Cs2—O3^vii^	139.0 (3)	Cs1^xv^—Se—Cs1^xvi^	114.79 (4)
O2^vii^—Cs2—O3^vii^	96.8 (4)	Cs1—Se—Cs1^xvi^	114.79 (4)
O4^viii^—Cs2—O3^vii^	48.2 (3)	Se—O1—W	129.4 (8)
O4^ix^—Cs2—O3^vii^	109.7 (4)	Se—O1—Cs1	122.3 (6)
O4^ii^—Cs2—O3^vii^	50.0 (3)	W—O1—Cs1	108.1 (5)
O3^vi^—Cs2—O3^vii^	83.0 (4)	Se—O1—Cs2	97.7 (5)
O3—Cs2—O3^vii^	83.0 (4)	W—O1—Cs2	91.2 (4)
O2—Cs2—O1^vii^	114.5 (4)	Cs1—O1—Cs2	83.4 (3)
O2^vi^—Cs2—O1^vii^	95.0 (4)	Se—O1—Cs2^xii^	97.7 (5)
O2^vii^—Cs2—O1^vii^	58.5 (4)	W—O1—Cs2^xii^	83.6 (4)
O4^viii^—Cs2—O1^vii^	47.1 (3)	Cs1—O1—Cs2^xii^	83.4 (3)
O4^ix^—Cs2—O1^vii^	143.4 (3)	Cs2—O1—Cs2^xii^	163.5 (5)
O4^ii^—Cs2—O1^vii^	83.8 (3)	W^xvii^—O2—Cs2	159.8 (9)
O3^vi^—Cs2—O1^vii^	97.0 (3)	W^xvii^—O2—Cs1	97.3 (5)
O3—Cs2—O1^vii^	127.0 (3)	Cs2—O2—Cs1	84.1 (4)
O3^vii^—Cs2—O1^vii^	45.0 (3)	W^xvii^—O2—Cs1^xvi^	103.6 (6)
O2—Cs2—O1	58.5 (4)	Cs2—O2—Cs1^xvi^	82.6 (3)
O2^vi^—Cs2—O1	114.5 (4)	Cs1—O2—Cs1^xvi^	152.0 (6)
O2^vii^—Cs2—O1	95.0 (4)	W^ix^—O3—W	148.6 (7)
O4^viii^—Cs2—O1	143.4 (3)	W^ix^—O3—Cs2	95.8 (5)
O4^ix^—Cs2—O1	83.8 (3)	W—O3—Cs2	101.6 (5)
O4^ii^—Cs2—O1	47.1 (3)	W^ix^—O3—Cs1^xiv^	101.4 (5)
O3^vi^—Cs2—O1	127.0 (3)	W—O3—Cs1^xiv^	106.7 (5)
O3—Cs2—O1	45.0 (3)	Cs2—O3—Cs1^xiv^	81.5 (2)
O3^vii^—Cs2—O1	97.0 (3)	W—O4—W^i^	135.7 (8)
O1^vii^—Cs2—O1	119.993 (7)	W—O4—Cs2^xii^	108.0 (5)
O2—Cs2—O1^vi^	95.0 (4)	W^i^—O4—Cs2^xii^	114.6 (5)
O2^vi^—Cs2—O1^vi^	58.5 (4)	W—O4—Cs1	99.0 (5)
O2^vii^—Cs2—O1^vi^	114.5 (4)	W^i^—O4—Cs1	96.3 (5)
O4^viii^—Cs2—O1^vi^	83.8 (3)	Cs2^xii^—O4—Cs1	84.8 (3)
O4^ix^—Cs2—O1^vi^	47.1 (3)		

Symmetry codes: (i) −*x*+*y*, −*x*+1, *z*; (ii) −*y*+1, *x*−*y*+1, *z*; (iii) *y*, −*x*+*y*+1, *z*−1/2; (iv) *x*−*y*, *x*, *z*−1/2; (v) −*x*+1, −*y*+1, *z*−1/2; (vi) −*y*+1, *x*−*y*, *z*; (vii) −*x*+*y*+1, −*x*+1, *z*; (viii) *x*+1, *y*, *z*; (ix) −*x*+*y*, −*x*, *z*; (x) *x*−*y*, *x*, *z*+1/2; (xi) −*y*, *x*−*y*, *z*; (xii) *x*−1, *y*, *z*; (xiii) −*x*, −*y*+1, *z*+1/2; (xiv) −*x*+1, −*y*+1, *z*+1/2; (xv) *x*−1, *y*−1, *z*; (xvi) *x*, *y*−1, *z*; (xvii) *y*, −*x*+*y*, *z*−1/2.

### Dirubidium tritungsten selenite (Rb2W3SeO123) Crystal data

**Table:**

Rb_2_W_3_SeO_12_	*D*_x_ = 6.004 Mg m^−^^3^
*M**_r_* = 993.40	Mo *K*α radiation, λ = 0.71073 Å
Hexagonal, *P*6_3_	Cell parameters from 19378 reflections
*a* = 7.2380 (1) Å	θ = 3.3–28.2°
*c* = 12.1115 (3) Å	µ = 43.49 mm^−^^1^
*V* = 549.50 (2) Å^3^	*T* = 293 K
*Z* = 2	Block, colourless
*F*(000) = 852	0.10 × 0.05 × 0.05 mm

### Dirubidium tritungsten selenite (Rb2W3SeO123) Data collection

**Table:**

Bruker APEXII CCD diffractometer	654 reflections with *I* > 2σ(*I*)
Radiation source: fine-focus sealed tube	*R*_int_ = 0.033
phi and ω scans	θ_max_ = 28.3°, θ_min_ = 1.7°
Absorption correction: multi-scan (SADABS; Krause *et al.*, 2015)	*h* = −9→8
*T*_min_ = 0.098, *T*_max_ = 0.220	*k* = −8→9
3497 measured reflections	*l* = −11→16
675 independent reflections	

### Dirubidium tritungsten selenite (Rb2W3SeO123) Refinement

**Table:**

Refinement on *F*^2^	*w* = 1/[σ^2^(*F*_o_^2^) + (0.0065*P*)^2^ + 2.2061*P*] where *P* = (*F*_o_^2^ + 2*F*_c_^2^)/3
Least-squares matrix: full	(Δ/σ)_max_ < 0.001
*R*[*F*^2^ > 2σ(*F*^2^)] = 0.018	Δρ_max_ = 0.90 e Å^−^^3^
*wR*(*F*^2^) = 0.033	Δρ_min_ = −1.14 e Å^−^^3^
*S* = 1.06	Extinction correction: SHELXL2018/3 (Sheldrick 2015b), Fc^*^=kFc[1+0.001xFc^2^λ^3^/sin(2θ)]^-1/4^
675 reflections	Extinction coefficient: 0.0058 (2)
57 parameters	Absolute structure: Flack *x* determined using 182 quotients [(*I*^+^)-(*I*^-^)]/[(*I*^+^)+(*I*^-^)] (Parsons *et al.*, 2013)
7 restraints	Absolute structure parameter: −0.08 (3)

### Dirubidium tritungsten selenite (Rb2W3SeO123) Special details

**Table:**

Geometry. All esds (except the esd in the dihedral angle between two l.s. planes) are estimated using the full covariance matrix. The cell esds are taken into account individually in the estimation of esds in distances, angles and torsion angles; correlations between esds in cell parameters are only used when they are defined by crystal symmetry. An approximate (isotropic) treatment of cell esds is used for estimating esds involving l.s. planes.
Refinement. Refined as a two-component twin

### Dirubidium tritungsten selenite (Rb2W3SeO123) Fractional atomic coordinates and isotropic or equivalent isotropic displacement parameters (Å^2^)

**Table:**

	*x*	*y*	*z*	*U*_iso_*/*U*_eq_	
Rb1	0.666667	0.333333	0.8979 (2)	0.0389 (9)	
Rb2	0.333333	0.666667	0.77053 (18)	0.0181 (6)	
W	0.80778 (6)	0.66034 (6)	0.60562 (3)	0.00482 (12)	
Se	1.000000	1.000000	0.83669 (14)	0.0057 (4)	
O1	0.8727 (15)	0.7523 (15)	0.7767 (6)	0.0106 (18)	
O2	0.5942 (14)	0.8028 (15)	0.9677 (7)	0.0106 (17)	
O3	0.7494 (12)	0.8784 (12)	0.5928 (8)	0.0084 (16)	
O4	0.9117 (13)	0.4617 (13)	0.6512 (6)	0.0069 (15)	

### Dirubidium tritungsten selenite (Rb2W3SeO123) Atomic displacement parameters (Å^2^)

**Table:**

	*U*^11^	*U*^22^	*U*^33^	*U*^12^	*U*^13^	*U*^23^
Rb1	0.0433 (13)	0.0433 (13)	0.0300 (16)	0.0216 (7)	0.000	0.000
Rb2	0.0202 (9)	0.0202 (9)	0.0139 (11)	0.0101 (4)	0.000	0.000
W	0.0036 (2)	0.0027 (2)	0.00779 (17)	0.00128 (19)	0.0005 (4)	−0.0003 (3)
Se	0.0062 (5)	0.0062 (5)	0.0047 (10)	0.0031 (3)	0.000	0.000
O1	0.016 (5)	0.008 (5)	0.010 (4)	0.008 (4)	0.003 (4)	0.003 (4)
O2	0.002 (4)	0.011 (5)	0.015 (4)	0.000 (4)	−0.003 (3)	−0.004 (4)
O3	0.007 (3)	0.006 (4)	0.012 (5)	0.004 (3)	0.000 (4)	0.001 (4)
O4	0.0065 (18)	0.0069 (19)	0.0077 (18)	0.0038 (12)	−0.0003 (12)	−0.0002 (12)

### Dirubidium tritungsten selenite (Rb2W3SeO123) Geometric parameters (Å, º)

**Table:**

Rb1—O1^i^	3.009 (9)	Rb2—O4^i^	3.012 (8)
Rb1—O1	3.009 (9)	Rb2—O3	3.382 (8)
Rb1—O1^ii^	3.009 (9)	Rb2—O3^vi^	3.382 (8)
Rb1—O4^ii^	3.360 (8)	Rb2—O3^vii^	3.382 (8)
Rb1—O4^i^	3.360 (8)	Rb2—W	3.9926 (11)
Rb1—O4	3.360 (8)	Rb2—W^vii^	3.9926 (11)
Rb1—O3^iii^	3.518 (9)	Rb2—W^vi^	3.9926 (11)
Rb1—O3^iv^	3.518 (9)	W—O2^x^	1.726 (8)
Rb1—O3^v^	3.518 (9)	W—O3	1.833 (8)
Rb1—W	4.094 (2)	W—O4^i^	1.860 (8)
Rb1—W^i^	4.094 (2)	W—O4	2.005 (8)
Rb1—W^ii^	4.094 (2)	W—O3^xi^	2.011 (8)
Rb2—O2	2.894 (8)	W—O1	2.155 (8)
Rb2—O2^vi^	2.894 (8)	Se—O1^ix^	1.714 (9)
Rb2—O2^vii^	2.894 (8)	Se—O1	1.714 (9)
Rb2—O4^viii^	3.012 (8)	Se—O1^xi^	1.714 (9)
Rb2—O4^ix^	3.012 (8)		
			
O1^i^—Rb1—O1	98.2 (2)	O4^ix^—Rb2—O3^vii^	111.8 (2)
O1^i^—Rb1—O1^ii^	98.2 (2)	O4^i^—Rb2—O3^vii^	48.78 (18)
O1—Rb1—O1^ii^	98.2 (2)	O3—Rb2—O3^vii^	83.8 (2)
O1^i^—Rb1—O4^ii^	51.2 (2)	O3^vi^—Rb2—O3^vii^	83.8 (2)
O1—Rb1—O4^ii^	88.0 (2)	O2—Rb2—W	89.89 (19)
O1^ii^—Rb1—O4^ii^	50.2 (2)	O2^vi^—Rb2—W	147.69 (19)
O1^i^—Rb1—O4^i^	50.2 (2)	O2^vii^—Rb2—W	113.3 (2)
O1—Rb1—O4^i^	51.2 (2)	O4^viii^—Rb2—W	114.82 (16)
O1^ii^—Rb1—O4^i^	88.0 (2)	O4^ix^—Rb2—W	75.92 (16)
O4^ii^—Rb1—O4^i^	46.7 (2)	O4^i^—Rb2—W	26.34 (16)
O1^i^—Rb1—O4	88.0 (2)	O3—Rb2—W	27.21 (13)
O1—Rb1—O4	50.2 (2)	O3^vi^—Rb2—W	105.50 (16)
O1^ii^—Rb1—O4	51.2 (2)	O3^vii^—Rb2—W	70.55 (14)
O4^ii^—Rb1—O4	46.7 (2)	O2—Rb2—W^vii^	147.69 (19)
O4^i^—Rb1—O4	46.7 (2)	O2^vi^—Rb2—W^vii^	113.3 (2)
O1^i^—Rb1—O3^iii^	130.6 (2)	O2^vii^—Rb2—W^vii^	89.89 (19)
O1—Rb1—O3^iii^	130.6 (2)	O4^viii^—Rb2—W^vii^	26.34 (16)
O1^ii^—Rb1—O3^iii^	71.3 (2)	O4^ix^—Rb2—W^vii^	114.82 (16)
O4^ii^—Rb1—O3^iii^	114.97 (19)	O4^i^—Rb2—W^vii^	75.92 (16)
O4^i^—Rb1—O3^iii^	159.4 (2)	O3—Rb2—W^vii^	105.50 (16)
O4—Rb1—O3^iii^	115.6 (2)	O3^vi^—Rb2—W^vii^	70.55 (14)
O1^i^—Rb1—O3^iv^	130.6 (2)	O3^vii^—Rb2—W^vii^	27.21 (13)
O1—Rb1—O3^iv^	71.3 (2)	W—Rb2—W^vii^	97.16 (4)
O1^ii^—Rb1—O3^iv^	130.6 (2)	O2—Rb2—W^vi^	113.3 (2)
O4^ii^—Rb1—O3^iv^	159.4 (2)	O2^vi^—Rb2—W^vi^	89.89 (19)
O4^i^—Rb1—O3^iv^	115.6 (2)	O2^vii^—Rb2—W^vi^	147.69 (19)
O4—Rb1—O3^iv^	115.0 (2)	O4^viii^—Rb2—W^vi^	75.92 (16)
O3^iii^—Rb1—O3^iv^	79.9 (2)	O4^ix^—Rb2—W^vi^	26.34 (16)
O1^i^—Rb1—O3^v^	71.3 (2)	O4^i^—Rb2—W^vi^	114.82 (16)
O1—Rb1—O3^v^	130.6 (2)	O3—Rb2—W^vi^	70.55 (14)
O1^ii^—Rb1—O3^v^	130.6 (2)	O3^vi^—Rb2—W^vi^	27.21 (13)
O4^ii^—Rb1—O3^v^	115.6 (2)	O3^vii^—Rb2—W^vi^	105.50 (16)
O4^i^—Rb1—O3^v^	114.97 (19)	W—Rb2—W^vi^	97.16 (4)
O4—Rb1—O3^v^	159.4 (2)	W^vii^—Rb2—W^vi^	97.16 (4)
O3^iii^—Rb1—O3^v^	79.9 (2)	O2^x^—W—O3	98.3 (4)
O3^iv^—Rb1—O3^v^	79.9 (2)	O2^x^—W—O4^i^	99.3 (4)
O1^i^—Rb1—W	76.74 (18)	O3—W—O4^i^	97.5 (3)
O1—Rb1—W	30.77 (17)	O2^x^—W—O4	93.8 (4)
O1^ii^—Rb1—W	79.82 (18)	O3—W—O4	166.2 (4)
O4^ii^—Rb1—W	57.35 (14)	O4^i^—W—O4	87.0 (5)
O4^i^—Rb1—W	26.63 (15)	O2^x^—W—O3^xi^	91.7 (5)
O4—Rb1—W	29.14 (14)	O3—W—O3^xi^	90.0 (4)
O3^iii^—Rb1—W	142.01 (14)	O4^i^—W—O3^xi^	165.5 (4)
O3^iv^—Rb1—W	102.05 (14)	O4—W—O3^xi^	83.0 (3)
O3^v^—Rb1—W	138.07 (13)	O2^x^—W—O1	172.2 (4)
O1^i^—Rb1—W^i^	30.77 (17)	O3—W—O1	85.6 (4)
O1—Rb1—W^i^	79.82 (18)	O4^i^—W—O1	86.8 (3)
O1^ii^—Rb1—W^i^	76.74 (17)	O4—W—O1	81.6 (3)
O4^ii^—Rb1—W^i^	26.63 (14)	O3^xi^—W—O1	81.4 (4)
O4^i^—Rb1—W^i^	29.14 (15)	O2^x^—W—Rb2	123.2 (3)
O4—Rb1—W^i^	57.35 (14)	O3—W—Rb2	57.5 (3)
O3^iii^—Rb1—W^i^	138.07 (13)	O4^i^—W—Rb2	45.9 (2)
O3^iv^—Rb1—W^i^	142.01 (14)	O4—W—Rb2	120.2 (2)
O3^v^—Rb1—W^i^	102.05 (14)	O3^xi^—W—Rb2	133.0 (3)
W—Rb1—W^i^	51.57 (3)	O1—W—Rb2	64.6 (2)
O1^i^—Rb1—W^ii^	79.82 (18)	O2^x^—W—Rb1	135.6 (3)
O1—Rb1—W^ii^	76.73 (17)	O3—W—Rb1	118.2 (3)
O1^ii^—Rb1—W^ii^	30.77 (17)	O4^i^—W—Rb1	54.1 (2)
O4^ii^—Rb1—W^ii^	29.14 (15)	O4—W—Rb1	54.7 (2)
O4^i^—Rb1—W^ii^	57.35 (14)	O3^xi^—W—Rb1	111.4 (3)
O4—Rb1—W^ii^	26.63 (14)	O1—W—Rb1	45.6 (3)
O3^iii^—Rb1—W^ii^	102.05 (14)	Rb2—W—Rb1	66.84 (2)
O3^iv^—Rb1—W^ii^	138.07 (13)	O2^x^—W—Rb1^xii^	59.3 (3)
O3^v^—Rb1—W^ii^	142.01 (14)	O3—W—Rb1^xii^	53.8 (3)
W—Rb1—W^ii^	51.57 (3)	O4^i^—W—Rb1^xii^	70.4 (2)
W^i^—Rb1—W^ii^	51.57 (3)	O4—W—Rb1^xii^	139.6 (2)
O2—Rb2—O2^vi^	58.6 (3)	O3^xi^—W—Rb1^xii^	123.7 (2)
O2—Rb2—O2^vii^	58.6 (3)	O1—W—Rb1^xii^	127.9 (3)
O2^vi^—Rb2—O2^vii^	58.6 (3)	Rb2—W—Rb1^xii^	66.06 (5)
O2—Rb2—O4^viii^	153.0 (2)	Rb1—W—Rb1^xii^	123.06 (2)
O2^vi^—Rb2—O4^viii^	97.5 (2)	O2^x^—W—Rb2^xiii^	113.1 (3)
O2^vii^—Rb2—O4^viii^	99.5 (3)	O3—W—Rb2^xiii^	128.1 (3)
O2—Rb2—O4^ix^	97.5 (2)	O4^i^—W—Rb2^xiii^	115.7 (2)
O2^vi^—Rb2—O4^ix^	99.5 (2)	O4—W—Rb2^xiii^	39.5 (2)
O2^vii^—Rb2—O4^ix^	153.0 (2)	O3^xi^—W—Rb2^xiii^	50.7 (2)
O4^viii^—Rb2—O4^ix^	98.91 (18)	O1—W—Rb2^xiii^	59.5 (2)
O2—Rb2—O4^i^	99.5 (2)	Rb2—W—Rb2^xiii^	122.13 (5)
O2^vi^—Rb2—O4^i^	153.0 (2)	Rb1—W—Rb2^xiii^	64.26 (2)
O2^vii^—Rb2—O4^i^	97.5 (2)	Rb1^xii^—W—Rb2^xiii^	171.73 (3)
O4^viii^—Rb2—O4^i^	98.91 (18)	O1^ix^—Se—O1	103.3 (3)
O4^ix^—Rb2—O4^i^	98.91 (18)	O1^ix^—Se—O1^xi^	103.3 (3)
O2—Rb2—O3	95.1 (2)	O1—Se—O1^xi^	103.3 (3)
O2^vi^—Rb2—O3	138.5 (3)	Se—O1—W	130.6 (5)
O2^vii^—Rb2—O3	137.5 (3)	Se—O1—Rb1	125.7 (4)
O4^viii^—Rb2—O3	111.8 (2)	W—O1—Rb1	103.7 (3)
O4^ix^—Rb2—O3	48.78 (18)	W^xiv^—O2—Rb2	159.3 (5)
O4^i^—Rb2—O3	51.09 (19)	W—O3—W^ix^	148.3 (5)
O2—Rb2—O3^vi^	137.5 (3)	W—O3—Rb2	95.3 (3)
O2^vi^—Rb2—O3^vi^	95.1 (2)	W^ix^—O3—Rb2	101.9 (3)
O2^vii^—Rb2—O3^vi^	138.5 (2)	W—O3—Rb1^xii^	101.4 (3)
O4^viii^—Rb2—O3^vi^	48.78 (18)	W^ix^—O3—Rb1^xii^	107.3 (3)
O4^ix^—Rb2—O3^vi^	51.09 (19)	Rb2—O3—Rb1^xii^	81.68 (17)
O4^i^—Rb2—O3^vi^	111.8 (2)	W^ii^—O4—W	134.3 (4)
O3—Rb2—O3^vi^	83.8 (2)	W^ii^—O4—Rb2^xiii^	107.7 (3)
O2—Rb2—O3^vii^	138.5 (2)	W—O4—Rb2^xiii^	115.5 (3)
O2^vi^—Rb2—O3^vii^	137.5 (3)	W^ii^—O4—Rb1	99.3 (3)
O2^vii^—Rb2—O3^vii^	95.1 (2)	W—O4—Rb1	96.2 (3)
O4^viii^—Rb2—O3^vii^	51.09 (19)	Rb2^xiii^—O4—Rb1	88.54 (18)

Symmetry codes: (i) −*y*+1, *x*−*y*, *z*; (ii) −*x*+*y*+1, −*x*+1, *z*; (iii) *y*, −*x*+*y*, *z*+1/2; (iv) *x*−*y*+1, *x*, *z*+1/2; (v) −*x*+1, −*y*+1, *z*+1/2; (vi) −*y*+1, *x*−*y*+1, *z*; (vii) −*x*+*y*, −*x*+1, *z*; (viii) *x*−1, *y*, *z*; (ix) −*x*+*y*+1, −*x*+2, *z*; (x) *x*−*y*+1, *x*, *z*−1/2; (xi) −*y*+2, *x*−*y*+1, *z*; (xii) −*x*+1, −*y*+1, *z*−1/2; (xiii) *x*+1, *y*, *z*; (xiv) *y*, −*x*+*y*+1, *z*+1/2.

### Dipotassium tritungsten selenite (K2W3SeO124) Crystal data

**Table:**

K_2_W_3_SeO_12_	*F*(000) = 1559
*M**_r_* = 900.66	*D*_x_ = 5.694 Mg m^−^^3^
Monoclinic, *P*2_1_/*n*	Mo *K*α radiation, λ = 0.71073 Å
*a* = 7.2310 (2) Å	Cell parameters from 9566 reflections
*b* = 11.4863 (4) Å	θ = 3.2–33.2°
*c* = 12.6486 (4) Å	µ = 37.09 mm^−^^1^
β = 90.096 (2)°	*T* = 293 K
*V* = 1050.56 (6) Å^3^	Block, colourless
*Z* = 4	0.08 × 0.05 × 0.02 mm

### Dipotassium tritungsten selenite (K2W3SeO124) Data collection

**Table:**

Bruker APEXII CCD diffractometer	3456 reflections with *I* > 2σ(*I*)
Radiation source: fine-focus sealed tube	*R*_int_ = 0.073
phi and ω scans	θ_max_ = 33.2°, θ_min_ = 3.2°
Absorption correction: multi-scan (SADABS; Krause *et al.*, 2015)	*h* = −11→11
*T*_min_ = 0.155, *T*_max_ = 0.524	*k* = −17→17
26562 measured reflections	*l* = −19→19
4026 independent reflections	

### Dipotassium tritungsten selenite (K2W3SeO124) Refinement

**Table:**

Refinement on *F*^2^	24 restraints
Least-squares matrix: full	*w* = 1/[σ^2^(*F*_o_^2^) + (0.0554*P*)^2^] where *P* = (*F*_o_^2^ + 2*F*_c_^2^)/3
*R*[*F*^2^ > 2σ(*F*^2^)] = 0.032	(Δ/σ)_max_ < 0.001
*wR*(*F*^2^) = 0.105	Δρ_max_ = 3.94 e Å^−^^3^
*S* = 1.15	Δρ_min_ = −3.11 e Å^−^^3^
4026 reflections	Extinction correction: SHELXL2018/3 (Sheldrick 2015b), Fc^*^=kFc[1+0.001xFc^2^λ^3^/sin(2θ)]^-1/4^
165 parameters	Extinction coefficient: 0.00176 (12)

### Dipotassium tritungsten selenite (K2W3SeO124) Special details

**Table:**

Geometry. All esds (except the esd in the dihedral angle between two l.s. planes) are estimated using the full covariance matrix. The cell esds are taken into account individually in the estimation of esds in distances, angles and torsion angles; correlations between esds in cell parameters are only used when they are defined by crystal symmetry. An approximate (isotropic) treatment of cell esds is used for estimating esds involving l.s. planes.
Refinement. Refined as a two-component twin

### Dipotassium tritungsten selenite (K2W3SeO124) Fractional atomic coordinates and isotropic or equivalent isotropic displacement parameters (Å^2^)

**Table:**

	*x*	*y*	*z*	*U*_iso_*/*U*_eq_	
K1	0.2501 (5)	0.4522 (3)	0.9115 (3)	0.0223 (6)	
K2	0.2548 (5)	0.0822 (3)	0.8952 (3)	0.0234 (7)	
W1	0.22893 (7)	0.79646 (4)	0.08433 (4)	0.00612 (10)	
W2	0.01720 (7)	0.75074 (4)	0.82437 (4)	0.00629 (11)	
W3	0.50663 (8)	0.75947 (4)	0.84420 (4)	0.00583 (11)	
Se	0.25041 (19)	0.49742 (10)	0.17926 (9)	0.0078 (2)	
O1	0.2536 (13)	0.6087 (8)	0.0913 (8)	0.0164 (19)	
O2	0.2387 (16)	0.9444 (8)	0.0619 (7)	0.016 (2)	
O3	0.0634 (14)	0.4225 (9)	0.1258 (9)	0.013 (2)	
O4	0.0680 (14)	0.8954 (9)	0.7993 (9)	0.014 (2)	
O5	0.4324 (14)	0.4206 (9)	0.1236 (8)	0.012 (2)	
O6	0.4376 (14)	0.8956 (9)	0.8021 (9)	0.015 (2)	
O7	0.0615 (15)	0.7625 (9)	0.9781 (9)	0.012 (2)	
O8	0.2548 (14)	0.6902 (7)	0.8102 (7)	0.0093 (16)	
O9	0.4378 (15)	0.7668 (9)	0.9828 (8)	0.0100 (19)	
O10	0.9354 (14)	0.7050 (10)	0.6903 (8)	0.014 (2)	
O11	0.7529 (15)	0.7855 (8)	0.8637 (7)	0.0096 (16)	
O12	0.0599 (14)	0.7924 (9)	0.1938 (8)	0.0110 (19)	

### Dipotassium tritungsten selenite (K2W3SeO124) Atomic displacement parameters (Å^2^)

**Table:**

	*U*^11^	*U*^22^	*U*^33^	*U*^12^	*U*^13^	*U*^23^
K1	0.0208 (14)	0.0264 (14)	0.0197 (14)	0.0030 (13)	−0.0020 (17)	0.0005 (13)
K2	0.0269 (17)	0.0211 (14)	0.0223 (16)	0.0007 (14)	−0.0005 (14)	0.0060 (12)
W1	0.00737 (19)	0.00678 (18)	0.00421 (17)	0.00057 (16)	−0.00022 (19)	0.00021 (17)
W2	0.0049 (2)	0.0084 (2)	0.0056 (2)	−0.0003 (2)	−0.00087 (18)	0.00082 (15)
W3	0.0047 (2)	0.0074 (2)	0.0054 (2)	0.00025 (19)	0.00025 (19)	−0.00006 (16)
Se	0.0090 (5)	0.0065 (4)	0.0078 (5)	0.0005 (4)	−0.0003 (5)	0.0003 (4)
O1	0.017 (2)	0.016 (2)	0.016 (2)	0.0001 (10)	0.0001 (10)	0.0005 (10)
O2	0.030 (6)	0.006 (4)	0.012 (4)	0.001 (4)	−0.005 (4)	0.002 (3)
O3	0.008 (4)	0.010 (4)	0.021 (5)	0.002 (4)	−0.009 (4)	−0.002 (4)
O4	0.013 (2)	0.014 (2)	0.014 (2)	0.0003 (10)	0.0000 (10)	0.0001 (10)
O5	0.011 (4)	0.010 (4)	0.016 (5)	0.003 (4)	0.003 (4)	0.003 (4)
O6	0.013 (5)	0.005 (4)	0.025 (6)	0.000 (4)	−0.002 (4)	0.008 (4)
O7	0.012 (2)	0.012 (2)	0.011 (2)	0.0002 (10)	−0.0004 (10)	0.0002 (10)
O8	0.003 (4)	0.010 (4)	0.015 (4)	0.003 (4)	−0.003 (4)	−0.001 (3)
O9	0.010 (2)	0.010 (2)	0.010 (2)	−0.0002 (10)	0.0005 (10)	−0.0004 (10)
O10	0.006 (4)	0.026 (6)	0.008 (5)	0.005 (4)	−0.004 (3)	0.000 (4)
O11	0.008 (4)	0.011 (4)	0.009 (4)	−0.002 (4)	−0.003 (4)	−0.004 (3)
O12	0.007 (4)	0.019 (5)	0.007 (4)	0.006 (4)	0.000 (3)	0.001 (4)

### Dipotassium tritungsten selenite (K2W3SeO124) Geometric parameters (Å, º)

**Table:**

K1—O3^i^	2.726 (10)	K2—W1^ii^	3.993 (4)
K1—O5^ii^	2.757 (11)	W1—O2	1.724 (9)
K1—O1^iii^	2.899 (10)	W1—O7^x^	1.849 (11)
K1—O5^iii^	3.009 (11)	W1—O12	1.849 (10)
K1—O8	3.019 (9)	W1—O10^xi^	2.005 (10)
K1—O4^iv^	3.046 (11)	W1—O9^x^	2.013 (11)
K1—O3^iii^	3.050 (12)	W1—O1	2.166 (9)
K1—O6^iv^	3.091 (12)	W2—O4	1.731 (11)
K1—Se^iii^	3.427 (4)	W2—O8	1.863 (10)
K1—Se^ii^	3.835 (4)	W2—O10^xii^	1.870 (10)
K1—Se^i^	3.839 (3)	W2—O7	1.975 (11)
K1—W2	3.975 (3)	W2—O11^xii^	2.016 (11)
K2—O2^v^	2.639 (9)	W2—O3^i^	2.167 (10)
K2—O6^vi^	2.780 (11)	W3—O6	1.726 (10)
K2—O4^vi^	2.809 (11)	W3—O11	1.822 (11)
K2—O10^vii^	2.861 (11)	W3—O9	1.825 (11)
K2—O8^iv^	2.879 (9)	W3—O12^xiii^	2.031 (10)
K2—O12^i^	2.918 (10)	W3—O8	2.032 (10)
K2—O9^viii^	3.213 (11)	W3—O5^ii^	2.153 (10)
K2—O7^ix^	3.316 (12)	Se—O1	1.695 (10)
K2—O11^viii^	3.408 (9)	Se—O5	1.735 (10)
K2—W2^iv^	3.767 (3)	Se—O3	1.739 (10)
K2—W1^i^	3.775 (4)		
			
O3^i^—K1—O5^ii^	112.6 (3)	O12—W1—K2^i^	49.0 (3)
O3^i^—K1—O1^iii^	79.3 (3)	O10^xi^—W1—K2^i^	130.3 (3)
O5^ii^—K1—O1^iii^	78.0 (3)	O9^x^—W1—K2^i^	143.4 (3)
O3^i^—K1—O5^iii^	125.5 (3)	O1—W1—K2^i^	116.2 (3)
O5^ii^—K1—O5^iii^	81.0 (3)	O2—W1—K2^ii^	68.2 (4)
O1^iii^—K1—O5^iii^	51.1 (3)	O7^x^—W1—K2^ii^	137.1 (4)
O3^i^—K1—O8	57.2 (3)	O12—W1—K2^ii^	125.4 (3)
O5^ii^—K1—O8	56.1 (3)	O10^xi^—W1—K2^ii^	42.7 (3)
O1^iii^—K1—O8	76.8 (3)	O9^x^—W1—K2^ii^	53.0 (3)
O5^iii^—K1—O8	118.8 (3)	O1—W1—K2^ii^	105.6 (3)
O3^i^—K1—O4^iv^	108.8 (3)	K2^i^—W1—K2^ii^	137.13 (9)
O5^ii^—K1—O4^iv^	67.2 (3)	O2—W1—K2^xiv^	26.6 (3)
O1^iii^—K1—O4^iv^	144.7 (3)	O7^x^—W1—K2^xiv^	76.9 (3)
O5^iii^—K1—O4^iv^	124.4 (3)	O12—W1—K2^xiv^	119.5 (3)
O8—K1—O4^iv^	79.5 (3)	O10^xi^—W1—K2^xiv^	111.4 (3)
O3^i^—K1—O3^iii^	81.0 (3)	O9^x^—W1—K2^xiv^	74.1 (3)
O5^ii^—K1—O3^iii^	124.9 (3)	O1—W1—K2^xiv^	145.5 (3)
O1^iii^—K1—O3^iii^	51.3 (3)	K2^i^—W1—K2^xiv^	77.64 (8)
O5^iii^—K1—O3^iii^	52.3 (2)	K2^ii^—W1—K2^xiv^	73.29 (8)
O8—K1—O3^iii^	118.9 (3)	O4—W2—O8	98.3 (4)
O4^iv^—K1—O3^iii^	161.2 (3)	O4—W2—O10^xii^	99.8 (5)
O3^i^—K1—O6^iv^	66.2 (3)	O8—W2—O10^xii^	95.6 (4)
O5^ii^—K1—O6^iv^	109.6 (3)	O4—W2—O7	94.6 (5)
O1^iii^—K1—O6^iv^	145.0 (3)	O8—W2—O7	88.4 (4)
O5^iii^—K1—O6^iv^	160.9 (3)	O10^xii^—W2—O7	164.3 (5)
O8—K1—O6^iv^	79.9 (3)	O4—W2—O11^xii^	93.2 (4)
O4^iv^—K1—O6^iv^	51.6 (3)	O8—W2—O11^xii^	166.7 (4)
O3^iii^—K1—O6^iv^	124.0 (3)	O10^xii^—W2—O11^xii^	88.9 (4)
O3^i^—K1—Se^iii^	95.2 (2)	O7—W2—O11^xii^	84.0 (4)
O5^ii^—K1—Se^iii^	94.6 (2)	O4—W2—O3^i^	172.6 (4)
O1^iii^—K1—Se^iii^	29.60 (19)	O8—W2—O3^i^	86.2 (4)
O5^iii^—K1—Se^iii^	30.4 (2)	O10^xii^—W2—O3^i^	85.5 (4)
O8—K1—Se^iii^	106.4 (2)	O7—W2—O3^i^	79.6 (4)
O4^iv^—K1—Se^iii^	153.9 (2)	O11^xii^—W2—O3^i^	81.6 (4)
O3^iii^—K1—Se^iii^	30.43 (19)	O4—W2—K2^xv^	105.4 (4)
O6^iv^—K1—Se^iii^	153.6 (2)	O8—W2—K2^xv^	48.1 (3)
O3^i^—K1—Se^ii^	130.7 (3)	O10^xii^—W2—K2^xv^	47.6 (3)
O5^ii^—K1—Se^ii^	24.1 (2)	O7—W2—K2^xv^	133.7 (3)
O1^iii^—K1—Se^ii^	97.7 (2)	O11^xii^—W2—K2^xv^	134.4 (3)
O5^iii^—K1—Se^ii^	82.7 (2)	O3^i^—W2—K2^xv^	82.0 (3)
O8—K1—Se^ii^	74.0 (2)	O4—W2—K1	142.1 (3)
O4^iv^—K1—Se^ii^	50.4 (2)	O8—W2—K1	46.7 (3)
O3^iii^—K1—Se^ii^	134.5 (2)	O10^xii^—W2—K1	98.2 (3)
O6^iv^—K1—Se^ii^	100.5 (2)	O7—W2—K1	73.6 (3)
Se^iii^—K1—Se^ii^	105.89 (9)	O11^xii^—W2—K1	120.3 (3)
O3^i^—K1—Se^i^	23.8 (2)	O3^i^—W2—K1	40.7 (3)
O5^ii^—K1—Se^i^	131.1 (2)	K2^xv^—W2—K1	64.86 (8)
O1^iii^—K1—Se^i^	98.5 (2)	O4—W2—K1^xv^	40.9 (4)
O5^iii^—K1—Se^i^	134.1 (2)	O8—W2—K1^xv^	76.3 (3)
O8—K1—Se^i^	75.4 (2)	O10^xii^—W2—K1^xv^	68.4 (3)
O4^iv^—K1—Se^i^	100.3 (2)	O7—W2—K1^xv^	127.3 (3)
O3^iii^—K1—Se^i^	82.19 (19)	O11^xii^—W2—K1^xv^	117.0 (3)
O6^iv^—K1—Se^i^	50.05 (19)	O3^i^—W2—K1^xv^	146.4 (3)
Se^iii^—K1—Se^i^	105.76 (9)	K2^xv^—W2—K1^xv^	64.97 (7)
Se^ii^—K1—Se^i^	140.87 (10)	K1—W2—K1^xv^	120.66 (4)
O3^i^—K1—W2	31.2 (2)	O6—W3—O11	100.1 (4)
O5^ii^—K1—W2	81.4 (2)	O6—W3—O9	100.1 (5)
O1^iii^—K1—W2	71.7 (2)	O11—W3—O9	97.5 (5)
O5^iii^—K1—W2	122.4 (2)	O6—W3—O12^xiii^	91.8 (5)
O8—K1—W2	26.7 (2)	O11—W3—O12^xiii^	89.3 (4)
O4^iv^—K1—W2	97.2 (2)	O9—W3—O12^xiii^	165.0 (4)
O3^iii^—K1—W2	98.9 (2)	O6—W3—O8	91.8 (4)
O6^iv^—K1—W2	75.8 (2)	O11—W3—O8	165.4 (4)
Se^iii^—K1—W2	98.21 (8)	O9—W3—O8	88.6 (4)
Se^ii^—K1—W2	100.71 (8)	O12^xiii^—W3—O8	81.8 (4)
Se^i^—K1—W2	52.29 (5)	O6—W3—O5^ii^	171.1 (4)
O2^v^—K2—O6^vi^	84.1 (3)	O11—W3—O5^ii^	86.1 (4)
O2^v^—K2—O4^vi^	82.2 (3)	O9—W3—O5^ii^	85.3 (4)
O6^vi^—K2—O4^vi^	57.1 (3)	O12^xiii^—W3—O5^ii^	81.8 (4)
O2^v^—K2—O10^vii^	129.3 (4)	O8—W3—O5^ii^	81.2 (4)
O6^vi^—K2—O10^vii^	81.2 (3)	O6—W3—K1	135.8 (3)
O4^vi^—K2—O10^vii^	126.1 (3)	O11—W3—K1	124.0 (3)
O2^v^—K2—O8^iv^	168.0 (3)	O9—W3—K1	73.4 (3)
O6^vi^—K2—O8^iv^	87.8 (3)	O12^xiii^—W3—K1	91.6 (3)
O4^vi^—K2—O8^iv^	85.9 (3)	O8—W3—K1	45.4 (2)
O10^vii^—K2—O8^iv^	57.6 (3)	O5^ii^—W3—K1	38.9 (3)
O2^v^—K2—O12^i^	124.7 (4)	O6—W3—K2^xv^	95.1 (4)
O6^vi^—K2—O12^i^	126.0 (3)	O11—W3—K2^xv^	129.0 (3)
O4^vi^—K2—O12^i^	80.6 (3)	O9—W3—K2^xv^	127.2 (3)
O10^vii^—K2—O12^i^	102.8 (3)	O12^xiii^—W3—K2^xv^	41.6 (3)
O8^iv^—K2—O12^i^	54.6 (3)	O8—W3—K2^xv^	40.4 (3)
O2^v^—K2—O9^viii^	88.4 (3)	O5^ii^—W3—K2^xv^	75.9 (3)
O6^vi^—K2—O9^viii^	106.9 (3)	K1—W3—K2^xv^	61.03 (7)
O4^vi^—K2—O9^viii^	162.1 (3)	O6—W3—K2^viii^	88.1 (4)
O10^vii^—K2—O9^viii^	51.2 (3)	O11—W3—K2^viii^	54.1 (3)
O8^iv^—K2—O9^viii^	102.5 (3)	O9—W3—K2^viii^	47.8 (3)
O12^i^—K2—O9^viii^	117.2 (3)	O12^xiii^—W3—K2^viii^	142.7 (3)
O2^v^—K2—O7^ix^	84.6 (3)	O8—W3—K2^viii^	135.5 (3)
O6^vi^—K2—O7^ix^	161.5 (3)	O5^ii^—W3—K2^viii^	100.7 (3)
O4^vi^—K2—O7^ix^	106.7 (3)	K1—W3—K2^viii^	113.79 (7)
O10^vii^—K2—O7^ix^	117.3 (3)	K2^xv^—W3—K2^viii^	174.68 (8)
O8^iv^—K2—O7^ix^	100.8 (3)	O1—Se—O5	96.1 (5)
O12^i^—K2—O7^ix^	51.9 (3)	O1—Se—O3	97.5 (5)
O9^viii^—K2—O7^ix^	87.4 (3)	O5—Se—O3	100.4 (4)
O2^v^—K2—O11^viii^	63.4 (3)	O1—Se—K1^x^	57.7 (3)
O6^vi^—K2—O11^viii^	137.0 (3)	O5—Se—K1^x^	61.4 (3)
O4^vi^—K2—O11^viii^	135.9 (3)	O3—Se—K1^x^	62.7 (4)
O10^vii^—K2—O11^viii^	97.6 (3)	O1—Se—K1^ii^	71.1 (3)
O8^iv^—K2—O11^viii^	127.9 (3)	O5—Se—K1^ii^	40.5 (3)
O12^i^—K2—O11^viii^	96.4 (3)	O3—Se—K1^ii^	133.7 (4)
O9^viii^—K2—O11^viii^	48.8 (3)	K1^x^—Se—K1^ii^	74.10 (9)
O7^ix^—K2—O11^viii^	46.8 (3)	O1—Se—K1^i^	72.8 (3)
O2^v^—K2—W2^iv^	156.5 (3)	O5—Se—K1^i^	132.1 (4)
O6^vi^—K2—W2^iv^	82.8 (2)	O3—Se—K1^i^	39.3 (3)
O4^vi^—K2—W2^iv^	106.6 (2)	K1^x^—Se—K1^i^	74.24 (9)
O10^vii^—K2—W2^iv^	28.8 (2)	K1^ii^—Se—K1^i^	140.87 (10)
O8^iv^—K2—W2^iv^	28.8 (2)	Se—O1—W1	141.0 (6)
O12^i^—K2—W2^iv^	78.7 (2)	Se—O1—K1^x^	92.7 (4)
O9^viii^—K2—W2^iv^	76.9 (2)	W1—O1—K1^x^	125.8 (4)
O7^ix^—K2—W2^iv^	112.5 (2)	W1—O2—K2^xiv^	136.4 (5)
O11^viii^—K2—W2^iv^	115.99 (18)	Se—O3—W2^i^	123.4 (5)
O2^v^—K2—W1^i^	97.2 (3)	Se—O3—K1^i^	116.9 (5)
O6^vi^—K2—W1^i^	139.0 (2)	W2^i^—O3—K1^i^	108.1 (4)
O4^vi^—K2—W1^i^	82.3 (2)	Se—O3—K1^x^	86.8 (4)
O10^vii^—K2—W1^i^	124.7 (2)	W2^i^—O3—K1^x^	118.7 (4)
O8^iv^—K2—W1^i^	83.2 (2)	K1^i^—O3—K1^x^	99.0 (3)
O12^i^—K2—W1^i^	28.6 (2)	W2—O4—K2^xvi^	139.1 (5)
O9^viii^—K2—W1^i^	114.1 (2)	W2—O4—K1^xv^	117.3 (5)
O7^ix^—K2—W1^i^	29.32 (19)	K2^xvi^—O4—K1^xv^	90.3 (3)
O11^viii^—K2—W1^i^	76.0 (2)	Se—O5—W3^ii^	124.5 (5)
W2^iv^—K2—W1^i^	105.51 (8)	Se—O5—K1^ii^	115.3 (5)
O2^v^—K2—W1^ii^	101.5 (3)	W3^ii^—O5—K1^ii^	111.7 (4)
O6^vi^—K2—W1^ii^	81.5 (2)	Se—O5—K1^x^	88.2 (4)
O4^vi^—K2—W1^ii^	138.0 (2)	W3^ii^—O5—K1^x^	112.0 (4)
O10^vii^—K2—W1^ii^	28.4 (2)	K1^ii^—O5—K1^x^	99.0 (3)
O8^iv^—K2—W1^ii^	85.9 (2)	W3—O6—K2^xvi^	134.9 (6)
O12^i^—K2—W1^ii^	125.5 (2)	W3—O6—K1^xv^	125.9 (5)
O9^viii^—K2—W1^ii^	30.0 (2)	K2^xvi^—O6—K1^xv^	90.0 (3)
O7^ix^—K2—W1^ii^	115.2 (2)	W1^iii^—O7—W2	146.6 (6)
O11^viii^—K2—W1^ii^	78.6 (2)	W1^iii^—O7—K2^ix^	89.2 (4)
W2^iv^—K2—W1^ii^	57.20 (5)	W2—O7—K2^ix^	113.7 (4)
W1^i^—K2—W1^ii^	137.13 (9)	W2—O8—W3	131.3 (5)
O2—W1—O7^x^	96.6 (5)	W2—O8—K2^xv^	103.1 (4)
O2—W1—O12	100.1 (5)	W3—O8—K2^xv^	112.3 (4)
O7^x^—W1—O12	96.1 (4)	W2—O8—K1	106.6 (4)
O2—W1—O10^xi^	95.0 (5)	W3—O8—K1	106.0 (3)
O7^x^—W1—O10^xi^	166.0 (5)	K2^xv^—O8—K1	89.6 (3)
O12—W1—O10^xi^	89.5 (4)	W3—O9—W1^iii^	145.8 (6)
O2—W1—O9^x^	91.8 (5)	W3—O9—K2^viii^	107.3 (4)
O7^x^—W1—O9^x^	89.5 (4)	W1^iii^—O9—K2^viii^	96.9 (4)
O12—W1—O9^x^	166.1 (4)	W2^xvii^—O10—W1^xiii^	147.4 (6)
O10^xi^—W1—O9^x^	82.3 (4)	W2^xvii^—O10—K2^xviii^	103.6 (4)
O2—W1—O1	169.9 (4)	W1^xiii^—O10—K2^xviii^	109.0 (4)
O7^x^—W1—O1	82.7 (4)	W3—O11—W2^xvii^	149.4 (5)
O12—W1—O1	89.9 (4)	W3—O11—K2^viii^	100.2 (4)
O10^xi^—W1—O1	84.4 (4)	W2^xvii^—O11—K2^viii^	109.0 (4)
O9^x^—W1—O1	78.2 (4)	W1—O12—W3^xi^	146.7 (5)
O2—W1—K2^i^	71.7 (4)	W1—O12—K2^i^	102.4 (4)
O7^x^—W1—K2^i^	61.4 (3)	W3^xi^—O12—K2^i^	110.9 (4)

Symmetry codes: (i) −*x*, −*y*+1, −*z*+1; (ii) −*x*+1, −*y*+1, −*z*+1; (iii) *x*, *y*, *z*+1; (iv) −*x*+1/2, *y*−1/2, −*z*+3/2; (v) *x*, *y*−1, *z*+1; (vi) *x*, *y*−1, *z*; (vii) −*x*+3/2, *y*−1/2, −*z*+3/2; (viii) −*x*+1, −*y*+1, −*z*+2; (ix) −*x*, −*y*+1, −*z*+2; (x) *x*, *y*, *z*−1; (xi) *x*−1/2, −*y*+3/2, *z*−1/2; (xii) *x*−1, *y*, *z*; (xiii) *x*+1/2, −*y*+3/2, *z*+1/2; (xiv) *x*, *y*+1, *z*−1; (xv) −*x*+1/2, *y*+1/2, −*z*+3/2; (xvi) *x*, *y*+1, *z*; (xvii) *x*+1, *y*, *z*; (xviii) −*x*+3/2, *y*+1/2, −*z*+3/2.
