# Supplementary material for: Structural characterization of quaternary selenites of tungsten(VI), A 2W3SeO12 (A = NH4, Cs, Rb, K or Tl)
Source: Acta Crystallogr E Crystallogr Commun. 2021 Mar 19;77(Pt 4):406–11. doi: 10.1107/S2056989021002735 (PMC8025859; doi:10.1107/S2056989021002735)
Supplement: Supplementary file 3 [file e-77-00406-sup6.pdf]

Electronic supplementary information (ESI)

Structural characterization of quaternary selenites of tungsten(VI), $A_2W_3SeO_{12}$ (A = NH_4, Cs, Rb, K, Tl)

Vineela Balisetty and Kanamaluru Vidyasagar*

Department of Chemistry, Indian Institute of Technology Madras, Chennai – 600036, India
**E.mail address:* kvsagar@iitm.ac.in

Details of other instruments employed

SEM and EDAX study

SEM images and EDAX data of single crystals of compounds **1-5** were recorded on FEI Quanta-200 and Quanta-450 scanning electron microscopes equipped with EDS detector.

Spectroscopic studies

The diffuse reflectance spectra were recorded on powder samples of compounds **1-5** at room temperature, on a JASCO UV-visible spectrophotometer with a diffuse reflectance accessory, over 200-900 nm range. $BaSO_4$ was used as a 100% reflectance standard. The absorption data was calculated (Kubelka & Munk 1931), using the Kubelka−Munk equation: $\alpha/S = (1 - R)^2/2R$, where R is the reflectance and α and S are the absorption and scattering coefficients, respectively.

Thermal studies

Thermogravimetric analytical (TGA) and Differential scanning calorimetry (DSC) data for compounds **3-5** were collected on 'Perkin-Elmer' Q-series TG and SDT Q600 V20.9 Build-20 instruments, respectively. The samples were heated to about 750 ºC at a rate of 20 ºC/min and cooled to about 120 ºC at the rate of 10 ºC/min under dynamic N_2 gas.

.

SUPPLEMENTARY TABLES

Table S1. Net dipole moments of WO_6 octahedra and SeO_3 pyramids and octahedral distortion of WO_6 octahedra of $A_2W_3SeO_{12}$ (A = NH_4(**1**), Cs(**2**), Rb(**3**) and K(**4α**)) compounds.

Table S2. Net dipole moment of WO_6 and TeO_3 polyhedra of $Rb_2W_3SeO_{12}$(**3**) compound.

Table S3. Net dipole moment of WO_6 and TeO_3 polyhedra of $K_2W_3SeO_{12}$(**4α**) compound.

Table S4. Quantities of reactants employed for the syntheses of $A_2W_3SeO_{12}$ (A = NH_4(**5**), Cs(**6**), Rb(**7**), K(**8α**) and Tl(**9**)) compounds.

SUPPLEMENTARY FIGURES

Figure S1a. Simulated (red) and observed (blue) powder XRD patterns of $A_2W_3SeO_{12}$ (A = NH_4(**1**) and Cs(**2**)) compounds.

Figure S1b. Simulated (red) and observed (blue) powder XRD patterns of $A_2W_3SeO_{12}$ (A = Rb(**3**), K(**4α**) and Tl(**5**)) compounds. Unidentified reflections are marked with asterisk.

Figure S2. ORTEP diagram of coordinations of A^+ ions of $A_2W_3SeO_{12}$ (A = NH_4(**1**), Cs(**2**), Rb(**3**) and K(**4α**)) compounds.

Figure S3. Thermal analysis of $A_2W_3SeO_{12}$ (A = Rb(**3**), K(**4α**) and Tl(**5**)) compounds.

Figure S4. Scanning electron microscope images of $A_2W_3SeO_{12}$ (A = NH_4(**1**), Cs(**2**), Rb(**3**), K(**4α**) and Tl(**5**)) compounds.

Table S1. Net dipole moments of WO_6 octahedra and SeO_3 pyramids and octahedral distortion of WO_6 octahedra of $A_2W_3SeO_{12}$ (A = NH_4(**1**), Cs(**2**), Rb(**3**) and K(**4α**)) compounds.

**Compound**	**WO_6 octahedra**	**Dipole moment (D)**	**Δd**	**TeO_3/ SeO_3 polyhedra**	**Dipole moment (D)**
$(NH_4)_2W_3SeO_{12}$**(1)**	WO_6	1.52	0.73	SeO_3	6.95
$Cs_2W_3SeO_{12}$**(2)**	WO_6	1.85	0.78	SeO_3	5.73
$Rb_2W_3SeO_{12}$**(3)**	WO_6	1.28	0.77	SeO_3	6.40
$K_2W_3SeO_{12}$(**4α**)	W(1)O_6	1.84	0.78	SeO_3	9.13
	W(2)O_6	1.30	0.71		
	W(3)O_6	0.79	0.86		

Table S2. Net dipole moment of WO_6 and SeO_3 polyhedra of $Rb_2W_3SeO_{12}$(**3**) compound.

Unit Cell Parameters					
a (Å)	7.238	α (°)	90	V (Å^3)	549.5
b(Å)	7.238	β (°)	90		
c (Å)	12.1115	γ (°)	120		

Atom	Bond Valence Charge	Number of Protons	Number of Electrons	Fractional Co-ordinates			Cartesian Co-ordinates			W-O Distance (Å)	W-O Bond length (Å)
				xf	yf	zf	xc	yc	zc		
W	6.2	74	67.800	0.80780	0.66030	0.60560	3.45723070	4.13895312	7.33475774		
O1	-0.526	8	8.526	0.87260	0.75220	0.77670	3.59366700	4.71500915	9.40704481	2.15518656	2.155
O2	-1.685	8	9.685	0.79160	0.59450	0.46780	3.57810530	3.72649952	5.66578545	1.72342599	1.723
O3	-0.776	8	8.776	1.12150	0.87100	0.59280	4.96526800	5.45968222	7.17972983	2.01060575	2.011
O3	-1.255	8	9.255	0.74950	0.87850	0.59280	2.24558950	5.50669441	7.17972983	1.83380050	1.834
O4	-1.17	8	9.170	0.53830	0.45000	0.65120	2.26766540	2.82073134	7.88704465	1.85951475	1.86
O4	-0.788	8	8.788	0.91170	0.46170	0.65120	4.92799230	2.89407036	7.88704465	2.00446841	2.005

Unit Vector (W-O)			C_Grav (Å)	C_ Charge (Å)	µ_ Debye	Dipole moment (W-O)		
UV xc	UV yf	UV zc				Dx	Dy	Dz
-0.06330603	-0.26728824	-0.96153489	0.21026210	0.24074523	11.16794546	-0.706998265	-2.9850605	-10.7383692
-0.07013623	0.23932191	0.96840381	0.16813912	0.21541435	17.58298042	-1.233203943	4.2079925	17.02742515
-0.75004127	-0.65688119	0.07710507	0.19615666	0.23042567	12.59608216	-9.447581502	-8.2741294	0.971221842
0.66072683	-0.74585065	0.08453913	0.17890737	0.22025597	15.29335945	10.10473298	-11.406562	1.292887351

0.63971813	0.70890633	-0.29700593	0.18141607	0.22153762	14.82314449	9.482634296	10.508221	-4.402561841	
-0.73374147	0.62105382	-0.27552787	0.19555789	0.23000037	12.66182607	-9.290506875	7.8636755	-3.488685955	Magnitude
						-1.090923308	-0.085863	0.661917351	1.278913871

Atom	Bond Valence Charge	Number of Protons	Number of Electrons	Fractional Co-ordinates			Cartesian Co-ordinates			Se-O Distance (Å)	Se-O Bond length (Å)
				xf	yf	zf	xc	yc	zc		
Se	3.888	34	30.112	1	1	0.8367	3.61900000	6.26829187	10.13373811		
O1	-1.296	8	9.296	0.8796	1.1274	0.7767	2.28648420	7.06687226	9.40704481	1.71505456	1.721
O1	-1.296	8	9.296	1.2478	1.1204	0.7767	4.97684880	7.02299421	9.40704481	1.71505456	1.721
O1	-1.296	8	9.296	0.8726	0.7522	0.7767	3.59366700	4.71500915	9.40704481	1.71505456	1.721

Unit Vector (Se-O)			C_Grav (Å)	C_ Charge (Å)	μ _ debye	Dipole moment (Se-O)			
UV xc	UV yf	UV zc				Dx	Dy	Dz	
0.77695243	-0.46562973	0.42371439	0.32667706	0.40456626	14.73339671	11.4471483	-6.86030747	6.242752252	
-0.79172338	-0.44004567	0.42371439	0.32667706	0.40456626	14.73339671	-11.6647747	-6.4833675	6.242752252	
0.01477096	0.90567540	0.42371439	0.32667706	0.40456626	14.73339671	0.217626394	13.343675	6.242752252	Magnitude
						-1.21569E-14	0	18.72825676	18.72825676

lone pair

Atom	Bond Valence Charge	Number of Protons	Number of Electron	Fractional Co-ordinates			Se-O distance (Å)	Unit Vector (Se-O)			UV
				xc	yc	zc		UV xc	UV yf	UV zc	

Unit Cell Parameters					
a (Å)	7.231	α (°)	90	V (Å^3)	1050.56
b(Å)	11.4863	β (°)	90.0959		
c (Å)	12.6486	γ (°)	90		

[illegible]	[illegible]	[illegible]	[illegible]	[illegible]	[illegible]						
[illegible]	[illegible]	[illegible]	[illegible]	[illegible]	[illegible]	0.00000000					
[illegible]	[illegible]	[illegible]	[illegible]	[illegible]	[illegible]	-0.72669330	1.71505456	-0.77695243	0.46562973	-0.42371439	1
O1	-1.296	8	9.296	1.55784880	0.75470254	-0.72669330	1.71505456	0.79172338	0.44004567	-0.42371439	1
O1	-1.296	8	9.296	-0.02533300	-1.55328273	-0.72669330	1.71505456	-0.01477096	-0.90567540	-0.42371439	1
centroid				0.00000000	0.00000000	-0.72669330					
lone pair	-2	2	4	-7.45554E-16	0	1.22	1.22000000	0.00000000	0.00000000	1.00000000	1

C_Grav (Å)	C_ Charge (Å)	µ_ Debye
0.32667706	0.40456626	14.73339671
0.32667706	0.40456626	14.73339671
0.32667706	0.40456626	14.73339671
0.06777778	0.14305816	12.32622933

Dipole moment (Se-O)			
Dx	Dy	Dz	
-11.4471483	6.860307468	-6.242752252	
11.6647747	6.483367497	-6.242752252	
-0.217626394	-13.34367497	-6.242752252	
-7.53268E-15	0	12.32622933	Magnitude
4.62427E-15	3.55271E-15	-6.402027422	6.40202742

Table S3. Net dipole moment of WO_6 and SeO_3 polyhedra of $K_2W_3SeO_{12}$(**4α**) compound.

Atom	Bond Valence Charge	Number of Protons	Number of Electrons	Fractional Co-ordinates			Cartesian Co-ordinates			W1-O Distance (Å)	W1-O Bond length (Å)
				xf	yf	zf	xc	yc	zc		
W1	6.161	74	67.839	0.22890	0.79650	0.08430	1.65339120	9.14883795	1.06627670		
O1	-0.51	8	8.510	0.25360	0.60870	0.09130	1.83184870	6.99171081	1.15481688	2.16630652	2.166
O2	-1.685	8	9.685	0.23870	0.94440	0.06190	1.72472922	10.84766172	0.78294813	1.72376518	1.724
O7	-1.205	8	9.205	0.06150	0.76250	-0.02190	0.44517014	8.75830375	-0.27700427	1.84843686	1.848
O9	-0.771	8	8.771	0.43780	0.76380	-0.01720	3.16609594	8.77323594	-0.21755586	2.01930150	2.019
O10	-0.788	8	8.788	0.43540	0.79500	0.19040	3.14434647	9.13160850	2.40829281	2.00605377	2.006
O12	-1.202	8	9.202	0.05990	0.79240	0.19380	0.42903399	9.10174412	2.45129804	1.84920321	1.849

Unit Vector (W1-O)			C_Grav (Å)	C_ Charge (Å)	μ_ Debye	Dipole moment (W1-O)			
UV xc	UV yf	UV zc				Dx	Dy	Dz	
-0.08237869	0.99576266	-0.04087149	0.21134698	0.24146051	11.03586286	-0.90911998	10.9891	-0.451052166	
-0.04138500	-0.98553085	0.16436610	0.16817221	0.21534835	17.55495912	-0.726512035	-17.300954	-2.885440225	
0.65364475	0.21127808	0.72671185	0.18033530	0.22084603	14.98131929	9.79246074	3.1652244	10.88710221	
-0.74912277	0.18600591	0.63578052	0.19700502	0.23118775	12.56994499	-9.416432052	2.338084	7.991726187	
-0.74322797	0.00858873	-0.66898312	0.19571256	0.23006513	12.63520304	-9.390836335	0.1085203	-8.45273755	
0.66209987	0.02546709	-0.74898277	0.18041007	0.22087418	14.96349978	9.907331296	0.3810768	-11.20740348	Magnitude
						-0.743108366	-0.3189481	1.653075431	1.840270715

Atom	Bond Valence Charge	Number of Protons	Number of Electrons	Fractional Co-ordinates			Cartesian Co-ordinates			W2-O Distance (Å)	W2-O Bond length (Å)
				xf	yf	zf	xc	yc	zc		
W2	6.079	74	67.921	0.01720	0.75070	0.82440	0.10691995	8.62276541	10.42750310		
O3	-0.509	8	8.509	-0.06340	0.57750	0.87420	-0.47695295	6.63333825	11.05740321	2.16691074	2.167
O4	-1.658	8	9.658	0.06800	0.89530	0.79940	0.47478403	10.28368439	10.11128818	1.73030856	1.73
O7	-0.855	8	8.855	0.06150	0.76250	0.97810	0.42399929	8.75830375	12.37159241	1.97443490	1.974
O8	-1.157	8	9.157	0.25490	0.69020	0.81020	1.82602928	7.92784426	10.24789303	1.86293104	1.863
O10	-1.135	8	9.135	-0.06460	0.70500	0.69030	-0.48173684	8.09784150	8.73132629	1.87058219	1.870
O11	-0.765	8	8.765	-0.24720	0.78550	0.86370	-1.80578846	9.02248865	10.92459295	2.01626647	2.016

Unit Vector (W2-O)			C_Grav (Å)	C_ Charge (Å)	μ_ Debye	Dipole moment (W2-O)			
UV xc	UV yf	UV zc				Dx	Dy	Dz	
0.26944945	0.91809373	-0.29069038	0.21140593	0.24124354	10.94634515	2.94948687	10.049771	-3.18199727	
-0.21260027	-0.95989757	0.18275059	0.16881059	0.21541036	17.35278342	-3.68920648	-16.656895	3.17123147	
-0.16059245	-0.06864665	-0.98463074	0.19262780	0.22772248	12.93326135	-2.07698414	-0.8878251	-12.73448674	
-0.92279815	0.37302570	0.09641263	0.18174937	0.22131944	14.63991173	-13.50968353	5.4610633	1.41147232	
0.31469175	0.28062061	0.90676412	0.18249582	0.22175779	14.52177662	4.56988336	4.0751098	13.16782604	
0.94863871	-0.19824921	-0.24653976	0.19670892	0.23045374	12.42122432	11.78325418	-2.4624979	-3.06232565	Magnitude
						0.0267500	-	-	1.2987911

69	0.4212737	1.228279827	53

Atom	Bond Valence Charge	Number of Protons	Number of Electrons	Fractional Co-ordinates			Cartesian Co-ordinates			W3-O Distance (Å)	W3-O Bond length (Å)
				xf	yf	zf	xc	yc	zc		
W3	6.24	74	67.760	0.50660	0.75950	0.84420	3.64535217	8.72384485	10.67794531		
O5	-0.527	8	8.527	0.56760	0.57940	0.87640	4.08576147	6.65516222	11.08523013	2.15390090	2.154
O6	-1.676	8	9.676	0.43760	0.89560	0.80210	3.14730446	10.28713028	10.14543939	1.72495664	1.725
O8	-0.733	8	8.733	0.25490	0.69020	0.81020	1.82602928	7.92784426	10.24789303	2.03187049	2.032
O9	-1.282	8	9.282	0.43780	0.76680	0.98280	3.14492509	8.80769484	12.43104081	1.82504847	1.825
O11	-1.293	8	9.293	0.75280	0.78550	0.86370	5.42521154	9.02248865	10.92459295	1.82151655	1.822
O12	-0.729	8	8.729	0.55990	0.70760	0.69380	4.03394857	8.12770588	8.77559637	2.03108846	2.031

Unit Vector (W3-O)			C_Grav (Å)	C_ Charge (Å)	µ_ Debye	Dipole moment (W3-O)		
UV xc	UV yf	UV zc				Dx	Dy	Dz
-0.20447055	0.96043538	-0.18909171	0.21013667	0.24075285	11.21095963	-2.292311065	10.767402	-2.119899571
0.28873057	-0.9062752	0.30870684	0.16828845	0.21554161	17.56365997	5.071165487	-15.9175	5.422022034

	0						1		
0.89539314	0.39175754	0.21165339	0.19823127	0.23197319	12.38889916	11.09293528	4.8534447	2.622152565	
0.27419934	-0.04594398	-0.96057476	0.17805351	0.21988136	15.46800689	4.241317227	-0.7106618	-14.85817704	
-0.97713049	-0.16395338	-0.13540785	0.17770893	0.21968455	15.52486554	-15.16981954	-2.5453542	-2.102188623	
-0.19132421	0.29350714	0.93661550	0.19815497	0.23178981	12.34893856	-2.362650919	3.6245017	11.56620732	Magnitude
						0.580636471	0.0718231	0.53011668	0.789506785

Atom	Bond Valence Charge	Number of Protons	Number of Electrons	Fractional Co-ordinates			Cartesian Co-ordinates			Se-O Distance (Å)	Se-O Bond length (Å)
				xf	yf	zf	xc	yc	zc		
Se	3.815	34	30.185	0.2504	0.4974	0.1793	1.80684647	5.71328562	2.26789338		
O1	-1.372	8	9.372	0.2536	0.6087	0.0913	1.83184870	6.99171081	1.15481688	1.69526853	1.695
O3	-1.215	8	9.215	0.0634	0.4225	0.1258	0.45578211	4.85296175	1.59119346	1.73880846	1.739
O5	-1.228	8	9.228	0.4324	0.4206	0.1236	3.12406768	4.83113778	1.56336655	1.73482408	1.735

Unit Vector (Se-O)			C_Grav (Å)	C_ Charge (Å)	µ _ debye	Dipole moment (Se-O)		
UV xc	UV yf	UV zc				Dx	Dy	Dz

-0.01474824	-0.75411368	0.65657829	0.32290829	0.40164969	14.95091222	-0.220499707	-11.2746874	9.816444334	
0.77700586	0.49477783	0.38917451	0.33120161	0.40667817	14.27412702	11.09108034	7.06252155	5.555126332	
-0.75928230	0.50849412	0.40610852	0.33044268	0.40618468	14.32905214	-10.87979563	7.28623871	5.819150104	Magnitude
						-0.009214996	3.07407282	21.19072077	21.41253501

lone pair

Atom	Bond Valence Charge	Number of Protons	Number of Electrons	Fractional Co-ordinates			Se-O distance (Å)	Unit Vector (Se-O)			UV
				xc	yc	zc		UV xc	UV yf	UV zc	
Se	3.815	34	30.185	0.00000000	0.00000000	0.00000000					
O1	-1.372	8	9.372	0.02500223	1.27842519	-1.11307651	1.69526853	0.01474824	0.75411368	-0.65657829	1
O3	-1.215	8	9.215	-1.35106436	-0.86032387	-0.67669992	1.73880846	-0.77700586	-0.49477783	-0.38917451	1
O5	-1.228	8	9.228	1.31722122	-0.88214784	-0.70452683	1.73482408	0.75928230	-0.50849412	-0.40610852	1
centroid				-0.00294697	-0.15468217	-0.83143442					
lone pair	-2	2	4	0.004251245	0.223141701	1.199412234	1.22000000	0.00348463	0.18290303	0.98312478	1

C_Grav (Å)	C_ Charge (Å)	μ_ debye
0.32290829	0.40164969	14.95091222
0.33120161	0.40667817	14.27412702

Dipole moment (Se-O)		
Dx	Dy	Dz
0.220499707	11.27468744	-9.816444334
-11.09108034	-7.062521552	-5.555126332

0.33044268	0.40618468	14.32905214
0.06777778	0.14275267	12.30248000

10.87979563	-7.286238708	-5.819150104	
0.042869555	2.250160914	12.09487297	Magnitude
0.052084551	-0.823911911	-9.095847798	9.13323551

	Compound	Reactants (g, mmol)				Amount of water	Temperature (°C) (duration (h))		Yield (%)
							Preheating in open air	Heating in evacuated and sealed quartz tube	
**Single crystal growth**	$(NH_4)_2W_3SeO_{12}$(**1**)	NH_4OH (6ml, 150.60)	H_2WO_4 (0.4360, 1.74)	SeO_2 (1.2, 10.80)	NH_4Cl (1, 18.35)	5ml	-	230 °C (96h)	~93 %
	$Cs_2W_3SeO_{12}$(**2**)	Cs_2CO_3 (0.1495, 0.46)	H_2WO_4 (0.3443, 1.38)	SeO_2 (0.2548, 2.30)	-	-	100 °C for 12 h	150°C(6h) + 600°C(24h)	-
	$Rb_2W_3SeO_{12}$(**3**)	Rb_2CO_3 (0.1156, 0.50)	WO_3 (0.3501, 1.50)	SeO_2 (0.1126, 1.00)	-	-	100 °C for 12 h	100 °C (2h) + 200 °C (24h) + 400 °C (24h) + 500 °C (24h)	-
	$K_2W_3SeO_{12}$(**4α**)	$(COOK)_2 \cdot H_2O$ (0.1057, 0.57)	H_2WO_4 (0.4310, 1.72)	@SeO_2 (0.0643, 0.57)	-	-	500 °C for 16 h	200 °C (24h) + 400 °C (24h) + 500 °C (24h)	-
	$Tl_2W_3SeO_{12}$(**5**)	Tl_2WO_4 (0.2666, 0.41)	WO_3 (0.1882, 0.81)	SeO_2 (0.0901, 0.81)	-	-	100 °C for 12 h	200 °C (20h) + 250 °C (16h) + 350 °C (16h) + 400 °C (48h) + 500 °C (24h) + 600 °C (18h)	~98 %

**Pow-der**	$Rb_2W_3SeO_{12}$(**3**)	Rb_2CO_3 (0.1156, 0.50)	H_2WO_4 (0.3773, 1.51)	@SeO_2 (0.2815, 2.51)	-	-	300 °C for 24 h	300 °C (24h) + 400 °C (12h) + 500 °C (12h)	~99 %

Table S4. Quantities of reactants employed for the syntheses of $A_2W_3SeO_{12}$ (A = NH_4(**1**), Cs(**2**), Rb(**3**), K(**4α**) and Tl(**5**)) compounds.

@SeO_2 was added after initial preheating.

SUPPLEMENTARY FIGURES

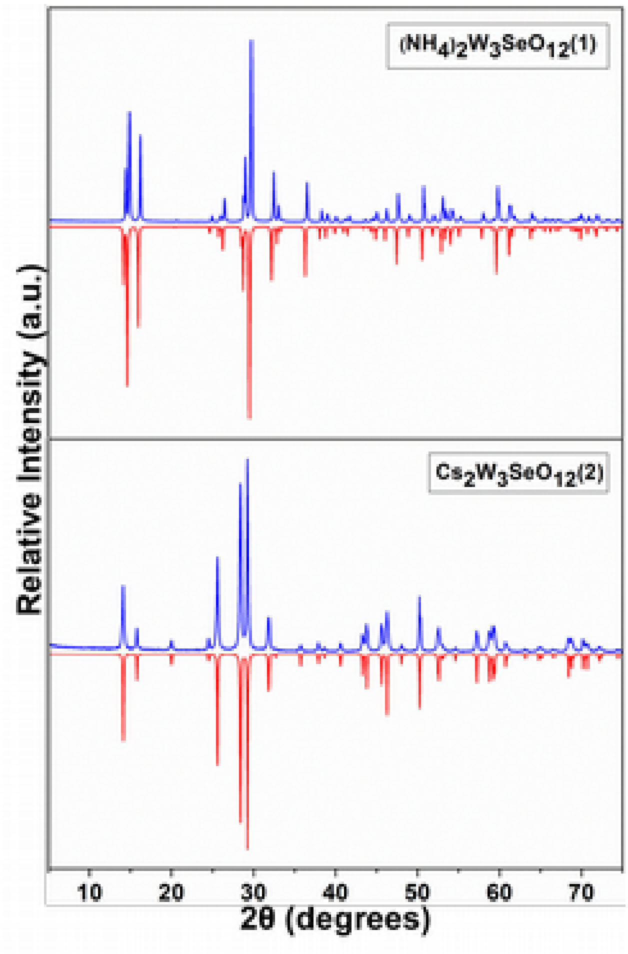

Figure S1a. Simulated (red) and observed (blue) powder XRD patterns of $A_2W_3SeO_{12}$ (A = NH_4(**1**) and Cs(**2**)) compounds.

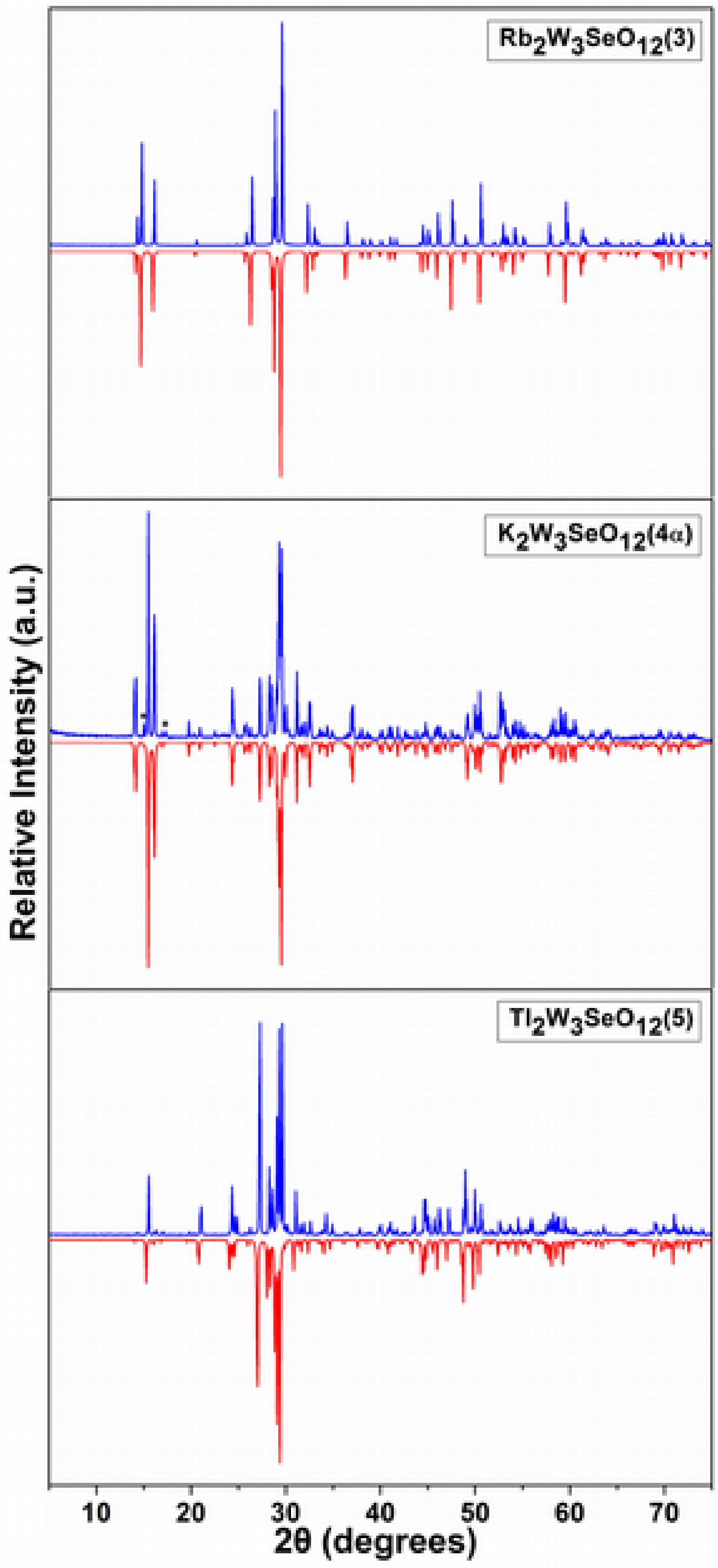

Figure S1b. Simulated (red) and observed (blue) powder XRD patterns of $A_2W_3SeO_{12}$ (A = Rb(**3**), K(**4α**) and Tl(**5**)) compounds. Unidentified reflections are marked with asterisk.

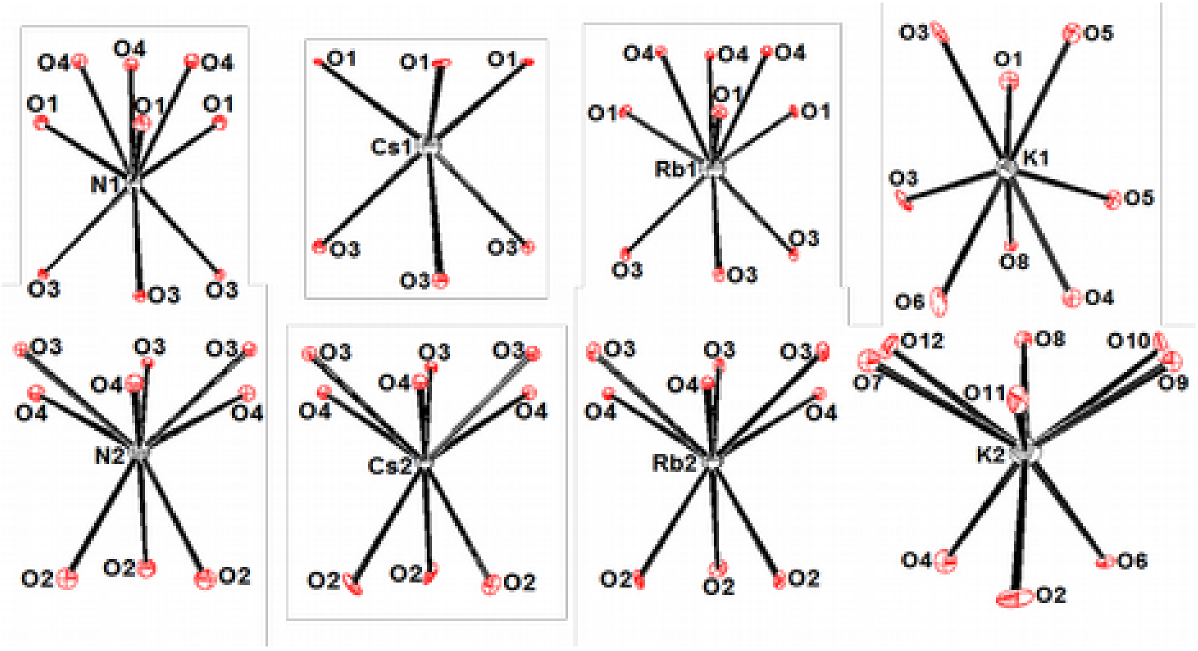

Figure S2. ORTEP diagram of coordinations of A^+ ions of $A_2W_3SeO_{12}$ (A = NH_4(**1**), Cs(**2**), Rb(**3**) and K(**4α**)) compounds.

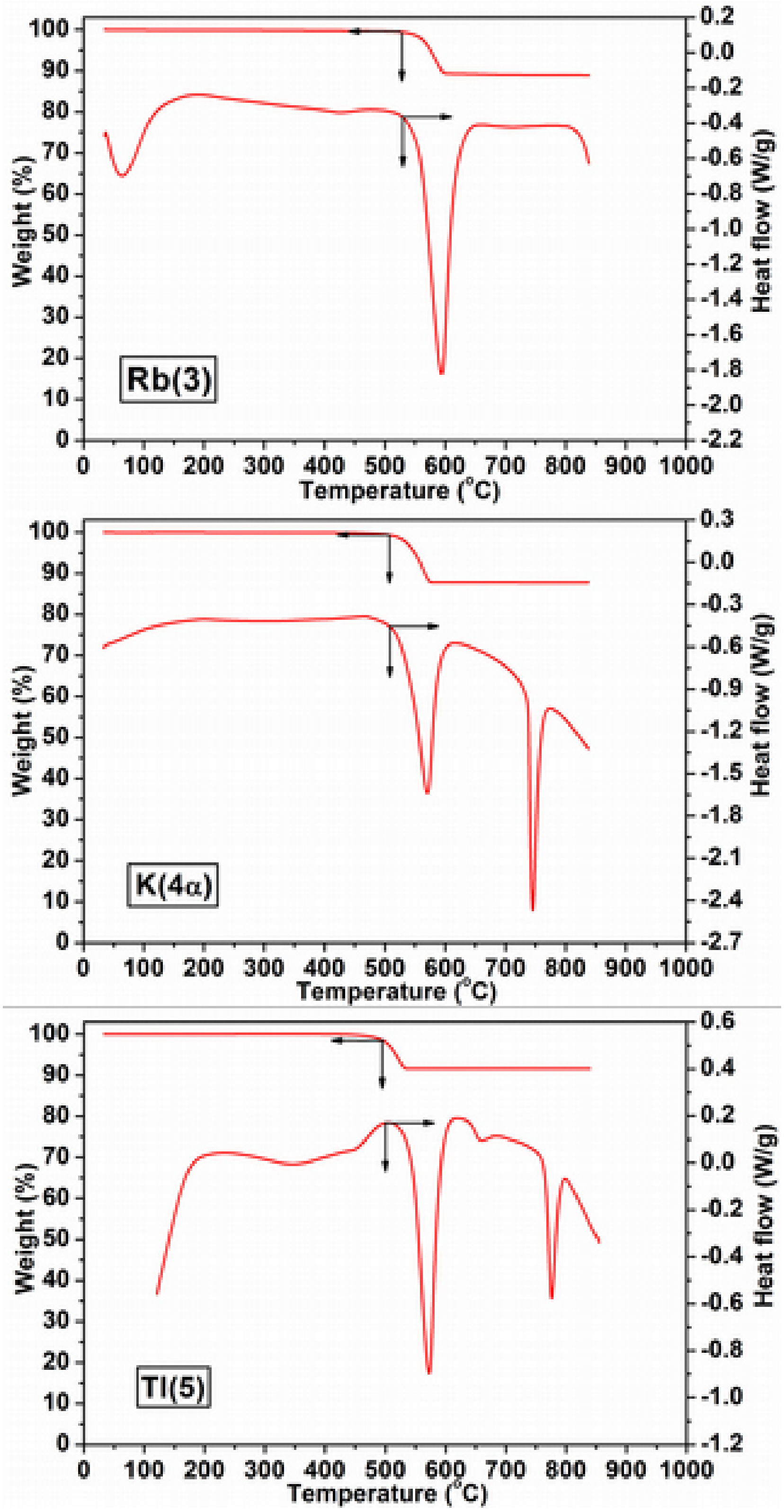

Figure S3. Thermal analysis of $A_2W_3SeO_{12}$ (A = Rb(**3**), K(**4α**) and Tl(**5**)) compounds.

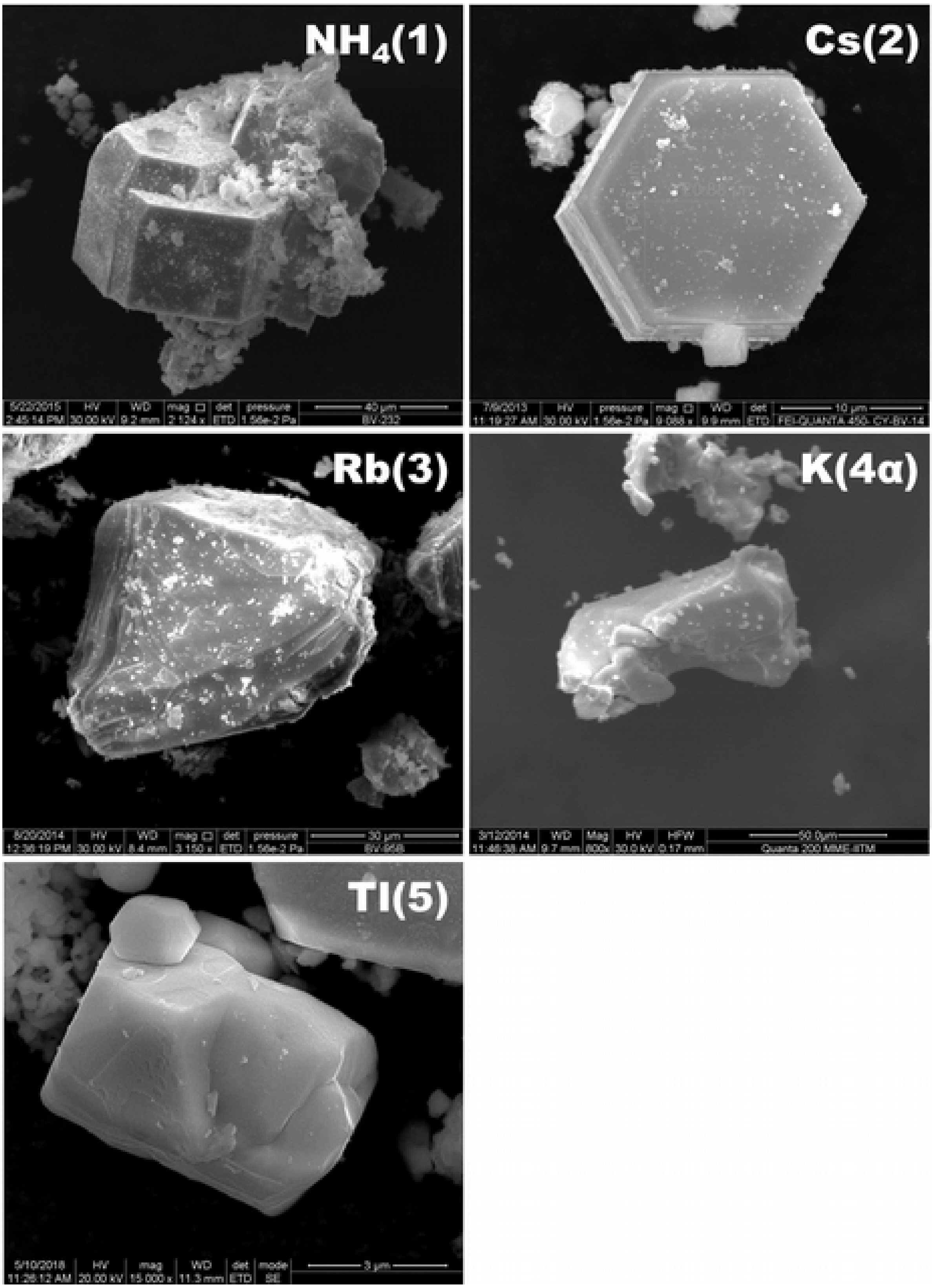

Figure S4. Scanning electron microscope images of $A_2W_3SeO_{12}$ (A = NH_4(**1**), Cs(**2**), Rb(**3**), K(**4α**) and Tl(**5**)) compounds.
